# The Conjugates of Indolo[2,3-*b*]quinoline as Anti-Pancreatic Cancer Agents: Design, Synthesis, Molecular Docking and Biological Evaluations †

**DOI:** 10.3390/ijms25052573

**Published:** 2024-02-22

**Authors:** Marcin Cybulski, Katarzyna Sidoryk, Magdalena Zaremba-Czogalla, Bartosz Trzaskowski, Marek Kubiszewski, Joanna Tobiasz, Anna Jaromin, Olga Michalak

**Affiliations:** 1 Pharmacy, Cosmetic Chemistry and Biotechnology Research Group, Łukasiewicz Research Network-Industrial Chemistry Institute, 01-793 Warsaw, Poland; marcin.cybulski@ichp.lukasiewicz.gov.pl (M.C.); k.sidoryk@pikralida.eu (K.S.); joanna.tobiasz@ichp.lukasiewicz.gov.pl (J.T.); 2 Department of Lipids and Liposomes, Faculty of Biotechnology, University of Wroclaw, 50-383 Wroclaw, Poland; magdalena.zaremba-czogalla@uwr.edu.pl (M.Z.-C.); anna.jaromin@uwr.edu.pl (A.J.); 3 Chemical and Biological Systems Simulation Lab, Center of New Technologies, University of Warsaw, 02-097 Warsaw, Poland; b.trzaskowski@cent.uw.edu.pl; 4 Pharmaceutical Analysis Laboratory, Łukasiewicz Research Network-Industrial Chemistry Institute, 01-793 Warsaw, Poland; marek.kubiszewski@ichp.lukasiewicz.gov.pl

**Keywords:** nature-derived molecules, biological activity, molecular modeling, organic synthesis, heterocyclic moieties

## Abstract

New amide conjugates of hydroxycinnamic acids (HCAs) and the known antineoplastic 5,11-dimethyl-5*H*-indolo[2,3-*b*]quinoline (DiMIQ), an analog of the natural alkaloid neocryptolepine, were synthesized and tested in vitro for anticancer activity. The compound 9-[((2-hydroxy)cinnamoyl)amino]-5,11-dimethyl-5*H*-indolo[2,3-*b*]quinoline (**2**), which contains the ortho-coumaric acid fragment, demonstrated dose-dependent effectiveness against both normal BxPC-3 and metastatic AsPC-1 pancreatic cancer cells. The IC_50_ values for AsPC-1 and BxPC-3 were 336.5 nM and 347.5 nM, respectively, with a selectivity index of approximately 5 for both pancreatic cancer cells compared to normal dermal fibroblasts. Conjugate **2** did not exhibit any hemolytic activity against human erythrocytes at the tested concentration. Computational studies were performed to predict the pharmacokinetic profile and potential mechanism of action of the synthesized conjugates. These studies focused on the ADME properties of the conjugates and their interactions with DNA, as well as DNA–topoisomerase alpha and beta complexes. All of the conjugates studied showed approximately one order of magnitude stronger binding to DNA compared to the reference DiMIQ, and approximately two orders of magnitude stronger binding to the topoisomerase II–DNA complex compared to DiMIQ. Conjugate **2** was predicted to have the strongest binding to the enzyme–DNA complex, with a Ki value of 2.8 nM.

## 1. Introduction

**Figure 1:**
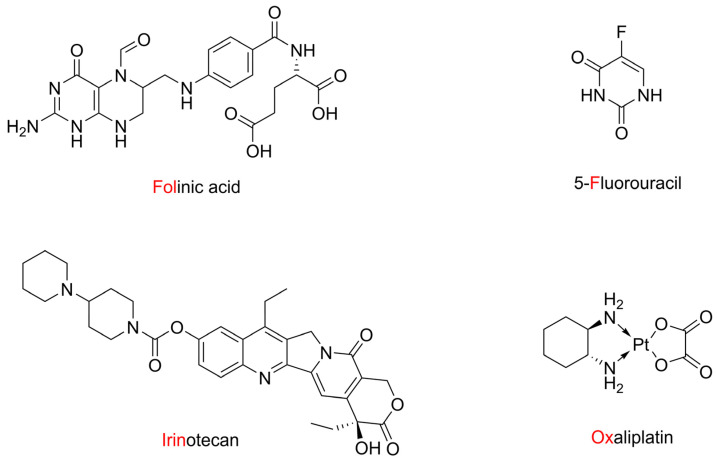
The structure of drugs included in FOLFIRINOX combination therapy against metastatic pancreatic cancer (also called FOLFOXIRI).

The use of multidrug therapies that entail a combination of chemotherapeutic agents with a different mechanism of action is common in oncological treatment, as cancer cells easily become resistant to individual drugs [1,2]. However, in some types of cancer, there is still a strong need to overcome limitations, even with targeted combination therapy. In the case of pancreatic cancer, which is currently the seventh most abundant form of cancer [3], the introduction of Folfirinox multidrug therapy (folinic acid, 5-fluorouracil, irinotecan, oxaliplatin: see Figure 1) in 2011 has not provided satisfactory results related to the survival rate. Similarly, the evaluation of combined gemcitabine therapies with other chemotherapeutic agents, such as topoisomerase inhibitors (capecitabine, pemetrexed), tyrosine kinase inhibitors (erlotinib, irinotecan), platinum compounds (cisplatin, oxaliplatin), taxanes (paclitaxel, docetaxel), in turn, against pancreatic cancer evidenced a lack of beneficial effects [4,5].

One of the directions related to the improvement of anticancer therapies is to find the combination of drugs that act synergistically [6]. Positive outcomes of synergistic drug therapies can result from both the accumulation of activities resulting from different and/or similar mechanisms of action, as well as better bioavailability. There are many examples of synergistic effects in preclinical studies: ceramide/docetaxel, cisplatin/olaparib, 5-fluorouracil/diosmetin [7,8,9,10,11]. However, in clinical analyses, some combinations of drugs exhibit additive, rather than synergistic therapeutic effects [12], or combination therapies afford predictable and clinically meaningful benefits, but without evidence of drug additivity and synergy effect [13].

**Figure 2:**
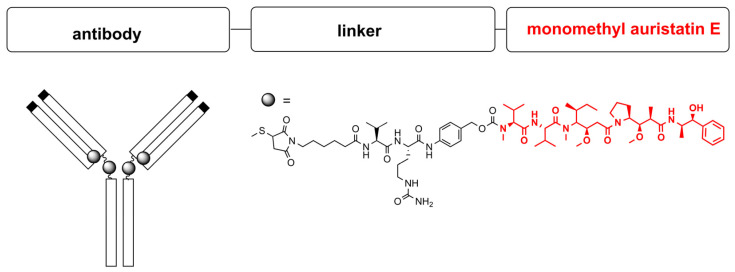
Schematic structure of registered drug Adcertis—the conjugate of brentuximab monoclonal antibody and monomethyl auristatin E antineoplastic agent [19].

The main obstacles to overcome are differences in the pharmacokinetics of drugs administered in specific time regimens, which could lead to suboptimal drug concentrations [14,15]. Following this observation, the different conjugates of anticancer compounds have been previously synthesized and biologically studied [16,17]. The most effective in anticancer therapy connections belong to antibody–drug conjugates (ADCs). Takeda’s brentuximab vedotin (Adcertis), which contains a monomethyl auristatin E antineoplastic agent conjugated via a linker to the chimeric monoclonal antibody brentuximab, has been the first registered anticancer drug in the class for the treatment of Hodgkin lymphoma (Figure 2) [18,19]. The other ADCs, such as Roches’s ado-trastuzumab emtansine (Kadcyla), registered for the treatment of HER2-positive breast cancer, consists of the monoclonal antibody trastuzumab, covalently bound to the microtubule inhibitor (a derivative of maytansine) by the thioether linker [20]. In 2021, the FDA approved another conjugate with the vedotin fragment (monomethyl auristatin E): tisotumab vedotin (Tivdak, Seagen/Genmab) to treat patients with metastatic or recurrent cervical cancer with disease progression [21]. To date, a total of eleven antibody–drug conjugates for cancer therapy have been approved by the FDA [22,23].

**Figure 3:**
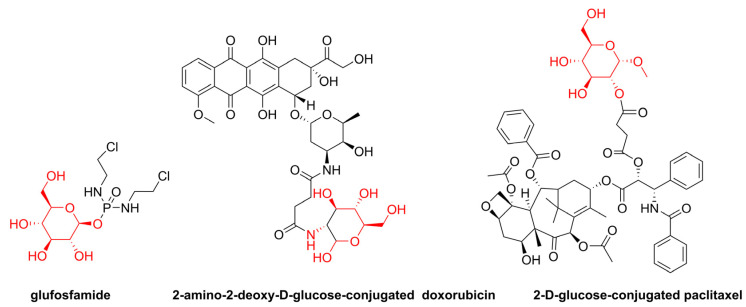
The examples of anticancer drug–sugar conjugates designed to enhance cancer targeting through GLUT transporter mechanism: glufosfamide, doxorubicin derivative, 2-D-glucose conjugated paclitaxel.

Folic acid conjugation is another approach to improve the uptake and targeting of cancer cells that express the folic acid receptor by chemotherapeutic agents. Vintafolide, a conjugate of folic acid and the vinca alkaloid desacetyl vinblastine hydrazide (DAVLBH), appeared to be effective in a Phase II trial but failed in a late-stage Phase III study against platinum-resistant ovarian cancer, due to the unsatisfactory progression-free survival period [24]. Another strategy to design anticancer compounds is to combine an anticancer drug with an attached glucose transporter substrate into a single structure to target cancer cells via the GLUT transporter mechanism (Figure 3) [25]. Glufosfamide was the first sugar conjugate of a series of similar compounds designed and evaluated as a cancer-targeting compound [26]. This compound is still under investigation whether it provides an additional survival benefit in patients with metastatic pancreatic cancer after a gemcitabine first-line regimen [27]. Similarly, the conjugate of doxorubicin topoisomerase II inhibitor with 2-amino-2-deoxyglucose moiety showed improved cytotoxicity when compared to doxorubicin itself [28]. The activity of 2-D-glucose conjugated paclitaxel has also been studied and discussed in the literature [29].

**Figure 4:**
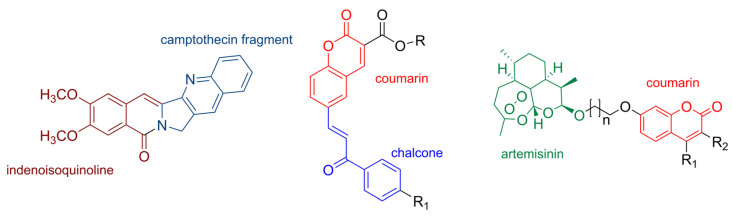
Examples of anticancer hybrid molecules: indenoisoquinoline–camptothecin, chalcone-coumarin, coumarin–artemisinin.

Preferential effects against cancer cells can also be enhanced by using a single bifunctional molecule as a new potential therapeutic agent that simultaneously targets different signaling pathways and distinct structures in cancer cells (Figure 4). The first strategy approach for hybrid molecules merges haptophoric groups selected from two drugs exhibiting the same mechanism of action [30]. Several anticancer hybrids designed following this concept have been synthesized, e.g., indenoisoquinoline–camptothecin hybrids as topoisomerase I inhibitors [31]. In the second strategy, anticancer hybrids possess substructures of drugs that act through different mechanism of action, such as chalcone-coumarin derivatives, which showed antiproliferative activity at the micromolar concentration [32], or other coumarin-containing hybrids [33].

Recently, intensive research has been focused on hydroxycinnamic acids (HCA) in order to find their new active hybrid molecules [34,35]. In addition to various pharmacological properties, hydroxycinnamic acids show interesting anticancer effects [36,37] related to the inhibition of cell proliferation [38]. HCAs affect the balance of intracellular reactive oxygen species (ROS), regulating lipid peroxidation and the permeability of the mitochondrial membrane. Ultimately, these effects may induce cell apoptosis [39]. In addition, HCAs also express antiangiogenic activity and suppress DNA methylation. The ability of caffeic acid (the most representative example compound of the HCA group) to bind to calf thymus DNA (Ct-DNA) has also been demonstrated [40]. Analysis of thermodynamic parameters has suggested that hydrogen bonds and van der Waals forces play a major role in the binding. Additional studies confirmed that caffeic acid interacts with the minor groove of Ct-DNA [40]. Our previous studies on caffeic acid derivatives have shown their cytotoxic activity against AsPC-1 and BxPC-3 pancreatic cancer cells and their low toxicity against normal NHDF cells (human skin fibroblasts) [36]. Moreover, some of the analogues of this compound, in combination with curcumin and/or carnosic acid, exhibit synergistic activity against leukemia cells [41].

Another molecule studied by our group, 5,11-dimethyl-5*H*-indolo[2,3-*b*]quinoline (DiMIQ **6**), the synthetic analog of natural alkaloid neocryptolepine, exhibits high cytotoxic activity against human mouth epidermal carcinoma KB cells at a concentration similar to that of doxorubicin [42,43]. DIMIQ (**6**) is capable of inducing DNA breaks in the BPV 1 episome in rat fibroblasts. Additionally, its DNA binding is evidenced by measuring the increase in calf thymus DNA denaturation temperature [42,43]. Our previously published results revealed that the improvement in the physicochemical and pharmacological properties of DiMIQ could be achieved by introducing selected amino acids or short peptides into its structure [44]. Such modifications result in favorable anticancer actions in vivo, with a relatively low hemolytic effect. The most interesting in vitro results have been obtained for a group of DiMIQ (**6**) analogues with guanidine or a guanyl-amino acid chain connected to the indolo[2,3-*b*]quinoline core. These modifications significantly improved cytoselectivity by increasing the cytotoxic effect against some cancer cell lines with respect to normal cells [45].

**Figure 5:**
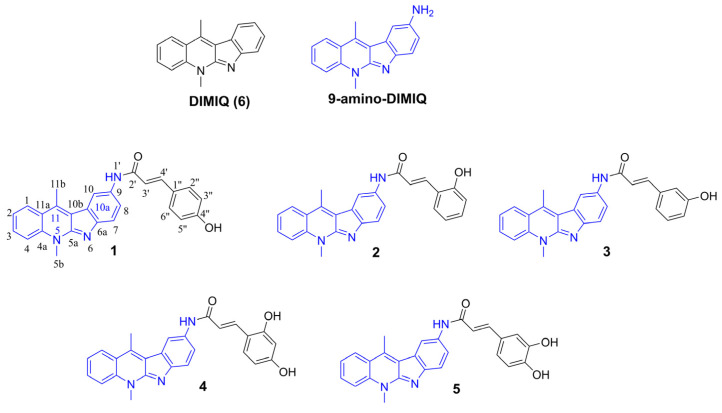
Chemical structures of DIMIQ (**6**), 9-amino-DiMIQ and HCA conjugates **1**–**5**.

The systematic increase in cancer incidence and still low survival rates [46] encourage intensive work in the search for new, effective therapies and novel diagnostic methods. Studying the bivalent compounds of chemotherapeutic agents is one of the promising approaches in this research area. Such hybrids can be classified into one of three basic types: linked, fused, or merged compounds [47,48,49] Therefore, in the present work, the synthesis of fused conjugates of a strong and highly documented cytostatic DNA intercalator, 5,11-dimethyl-5*H*-indolo[2,3-*b*]quinoline, with selected ROS-modulating hydroxycinnamic acids is reported (Figure 5). All analogues have been evaluated in vitro for their antiproliferative profile against cancer cell lines. The cytotoxicity of all compounds was determined by the MTT assay. To identify the structures of all isolated products, a detailed analysis of 1D and 2D NMR experiments were performed.

## 2. Results and Discussion

### 2.1. Chemistry

**Scheme 1:**
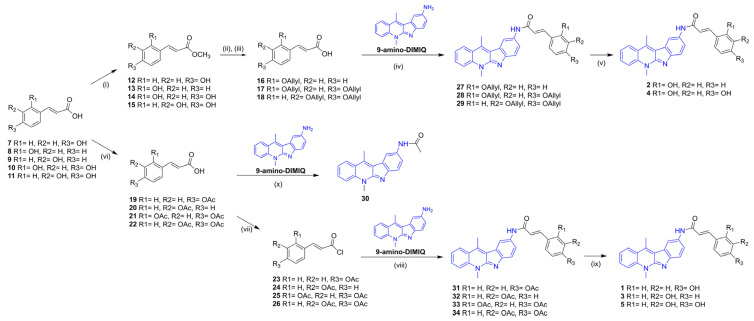
Synthesis of **1**–**5** conjugates. Reagents and reaction conditions: (**i**) CH_3_OH, CH_3_COCl, r.t.; (**ii**) allyl bromide, K_2_CO_3_, acetone, r.t.; (**iii**) NaOH, H_2_O/MeOH; (**iv**) TBTU, DIPEA, DMF, r.t.; (**v**) Et_3_SiH, Pd(PPh_3_)_4_, DMF, r.t.; (**vi**) pyridine, DMAP, (CH_3_CO)_2_O; (**vii**) SOCl_2_, DCM; (**viii**) pyridine, DCM, −20*–*−5 °C then r.t; (**ix**) K_2_CO_3_, DMF, r.t.; (**x**) DCC, DMAP, DMF, 0 °C, then r.t.

The effective synthesis of the designed conjugates required the use of protected hydroxycinnamic acids (HCAs) as building blocks for coupling with 9-amino-DiMIQ. To compare the efficiency of coupling reactions with an aromatic amino group and the effectiveness of the subsequent removal of protecting groups from the designed conjugates, the acetyl and allyl protected HCAs were selected as substrates (Scheme 1). These compounds were previously synthesized under standard reaction conditions. HCA O-acetates were prepared from the respective acids in the presence of acetic anhydride, pyridine, and 4-dimethylaminopyridine (DMAP) [50]. After workup with water dilution, acidification, filtration, and optional crystallization, the acetyl derivatives **19**–**22** were obtained, with yields of 60–95%. Synthesis of O-allyl HCA derivatives was completed in a three-step procedure comprising esterification with methyl alcohol (**12**–**15**) [51], allylation of HCA ester by allyl bromide in acetone with K_2_CO_3_, followed by hydrolysis of the product in basic conditions (NaOH aq./methanol) to obtain allyl-protected HCA **16**–**18** with a yield of 75 to 80%.

The coupling reactions were performed using typical reaction conditions with the selected agents: DCC, EDCI, or TBTU to obtain the most promising results for DCC/DMAP in the DMF system. The reactions were evidenced to run more efficiently with HCA derivatives containing the allyl-protecting group to give protected conjugates **27**–**29** with a yield of 68–75%. The coupling of 5,11-dimethyl-5*H*-indolo[2,3-b]quinolin-9-yl-amine with O-acetyl protected hydroxycinnamic acids resulted in the migration of the acetyl group to give N-(5,11-dimethyl-5*H*-indolo[2,3-b]quinolin-9-yl)acetamide (**30**) as the main reaction product.

Moreover, migration occurred much easier in reactions with protected dihydroxycinnamic acid substrates than with monohydroxycinnamic ones. Acetamide impurity **30** was isolated and characterized using the NMR technique (Scheme 1). The difficulties in obtaining peracetylated conjugates under peptide-type coupling conditions prompted us to search for another effective method to avoid impurity formation, as well as to obtain **31**–**34** compounds with acceptable yields. It was decided to apply respective acid chlorides for amide formation because of the small size of the chloride leaving group and their tendency to react rapidly. At first, the protocol described by Quéléver et al. [52], where acid and amine substrates react in the presence of POCl_3_ to first form the respective acid chloride in situ, was experimentally verified, without a positive result. Therefore, we converted the peracetylated acids **19**–**22** to the corresponding acid chlorides **23**–**26** in a standard procedure, with an excess of thionyl chloride in dichloromethane. After the reactions were completed, the excess of solvent and the chlorinating agent were evaporated. The crude acid chlorides were dissolved in dichloromethane (DCM), then evaporated to solid, dried under vacuum at room temperature, and finally used in the next step, without further purification. Then, Shimma et al.’s [53] protocol for the preparation of the capecitabine anticancer drug was applied. Solutions of acid chlorides **23**–**26** in dichloromethane were added dropwise to the suspension of 9-amino-DiMIQ in DCM and the pyridine solvent mixture at a temperature below −5 °C. Next, reactions were continued at room temperature. Chromatographic purifications led to expected products **31**–**34** with a yield of 35–58%. The next step was to test the effectiveness of unlocking both types of protection. Therefore, two model compounds were selected, namely **27**, with a protected hydroxyl group, and **28,** with two hydroxyl groups, to find acceptable reaction conditions for the removal of allylic protection. Test reactions were carried out in the presence of tetrakis(triphenylphosphine)palladium [54] or by using the reagent systems DMSO-I_2_ and DMSO-NaI [55,56]. The attempt with Pd(0) led to a complicated post-reaction mixture (TLC monitoring). Reaction with iodine/iodide at room temperature did not show any progress. When heated to 90 °C, followed by stirring for 3 h and leaving overnight at room temperature, the cleavage of conjugates to the respective O-allyl-hydroxycinnamic acids was observed. In the reaction with tetrakis(triphenylphosphine)-palladium, various conditions of deprotection were also verified, such as the solvents (methanol, DMF) and the molar excesses of the catalyst. Each time, the multispot image related to various decomposition products and without the main product was observed on TLC plates. The removal of the allyl group from compound **27** was achieved in the presence of triethylsilane, the free radical scavenger, with an unusual half of the equimolar amount of Pd(0) catalyst, to provide the product with a yield of 50%. The deallylation of compound **28** was performed under similar conditions, with the equimolar amount of catalyst. Despite the controlled course of the reaction, the removal of both allyl groups, leading to compound **4**, was achieved with an unsatisfactory yield of 34%. Therefore, further efforts have been focused on obtaining conjugates designed from peracetylated, rather than allyl derivatives. Although the deacetylations were carried out under mild basic conditions [50,57], with good yields of 78–84%, the formation of the acetamide by-product **30** was also detected. Finally, all five compounds **1**–**5** were converted into respective dihydrochlorides using the molar excess of 2 M gaseous HCl in ethyl acetate.

### 2.2. Anticancer Activity

**Figure 6:**
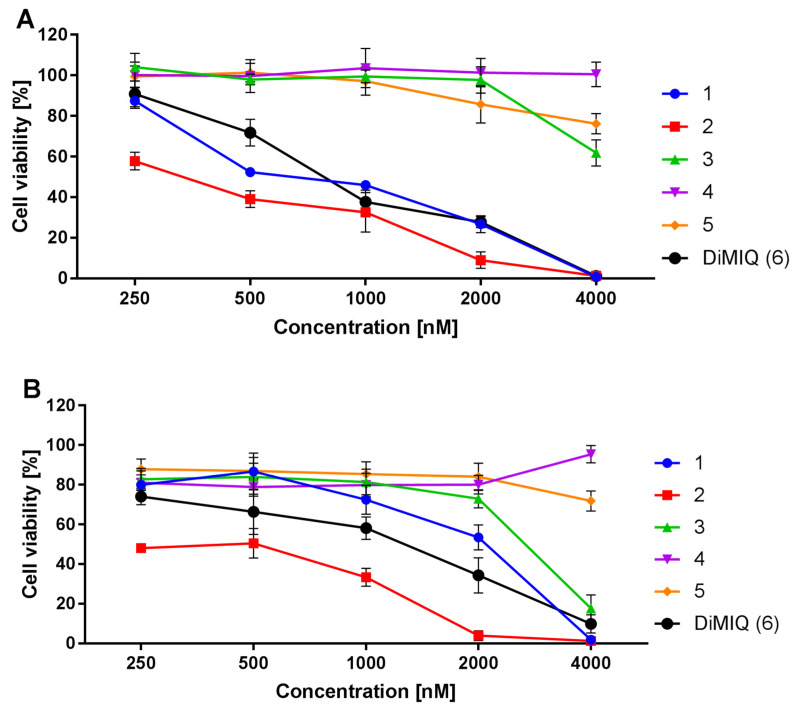
Cytotoxic effect of newly synthesized conjugates **1**–**5** and control DiMIQ (**6**), determined by the MTT assay, after a 72 h incubation period for BxPC-3 (**A**) and AsPC-1 (**B**) cell lines. The results were expressed as % of viability compared to the untreated control.

As part of preliminary biological studies, conjugates **1**–**5** and the reference compound 5,11-dimethyl-5*H*-indolo[2,3-b]quinoline (DiMIQ **6**) were examined for their cytotoxic activity using the MTT method adapted from Mosmann [58]. After their dissolution in DMSO (see Supplementary Materials, Figures S18 and S19), conjugates **1**–**5** were evaluated in vitro against metastatic AsPC-1 and primary BxPC-3 pancreatic cancer cell lines, MCF-7 breast cancer cell line, and HeLa cervical carcinoma cell line. All cells used belonged to adherent cell lines and grew as monolayers in 96-well plates (5000 cells per well) in our experiments. Conjugates in DMSO **1**–**5** were diluted in a culture medium to the appropriate concentration, then applied to cells, and incubated for 72 h. The untreated cells served as controls, and their viability was considered as 100%. In general, only two of the designed and synthesized compounds (namely **1** and **2**) showed similar or higher cytotoxic activities against both tested pancreatic cancer cell lines (BxPC-3 and AsPC-1) compared to the 5,11-dimethyl-5*H*-indolo[2,3-*b*]quinoline (DiMIQ **6**) (see Figure 6 and Table 1). When analyzing the results, it was evidenced that the cells reacted in a dose-dependent manner to the applied concentration range (250–4000 nM).

**Figure 7:**
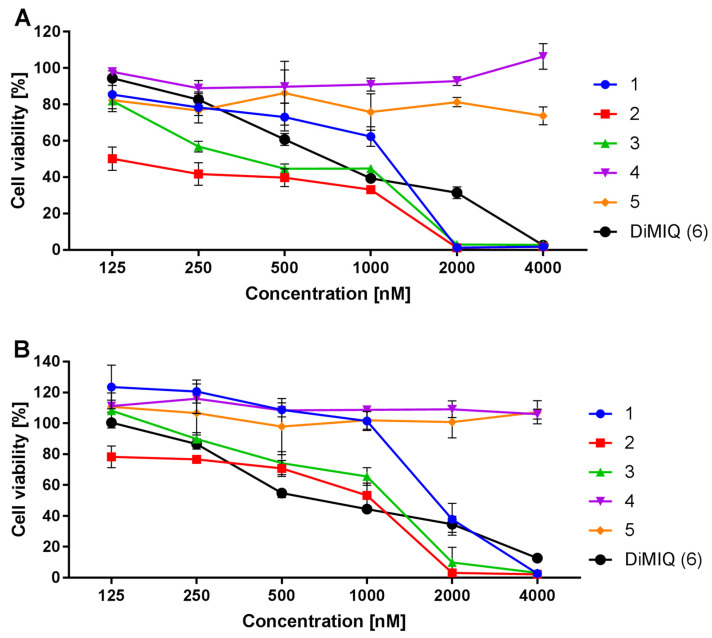
Cytotoxic effects of conjugates **1**–**5** and reference compound DiMIQ (**6**) on HeLa (**A**) and MCF-7 cells (**B**) after a 72 h incubation period. The results were expressed as a % of viability compared to the untreated control.

Dose-dependent inhibition of tumor cell growth was also observed in the hormone-dependent breast cancer line MCF-7 and the cervical cancer line HeLa (Figure 7). As in the case of pancreatic cancer cells, the best cytotoxic effects were observed for compounds **1** and **2**. Furthermore, compound **3**, less active against BxPC-3 and AsPC-1 cell lines than DiMIQ (**6**), showed significant point activity at 2000 nM against HeLa cells. At 4000 nM, its activity was comparable to that of DiMIQ. In the case of MCF-7, only conjugates **2** and **3** showed significant activity in inhibiting tumor cell growth.

### 2.3. Toxicity Effects on Normal Human NHDF Cells and Human Erythrocytes

**Figure 8:**
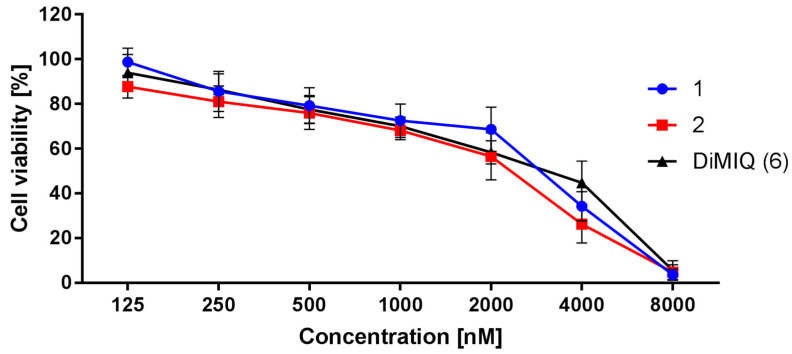
Cytotoxic effects of selected conjugates (**1** and **2**) and 5,11-dimethyl-5*H*-indolo[2,3-*b*]quinoline (**6**) on NHDF cells, determined by the MTT assay after 72 h of incubation. The results were expressed as % of viability compared to the untreated control.

Cancer chemotherapy is limited by its toxicity to normal cells. Therefore, traditional cancer drugs must be selective for cancer cells and exploit the proliferative advantage of cancer cells over normal ones. Although the results of the in vitro tests are of limited importance in terms of the clinical toxicity of chemo drugs, they are still represent the first characteristic of new compounds as anticancer therapeutics with a potential therapeutic window [59]. Therefore, normal human skin fibroblast NHDFs were used as model cells to evaluate the in vitro cytotoxicity of compounds **1**–**2** because these compounds have proved effective against cancer cell lines in our studies. After 72 h of incubation, compounds **1** and **2** influenced the viability of normal human cells to a lesser degree, compared to the viability of both cancer pancreatic lines. The calculated SI values of **2** were approximately two and three times higher than the SI of DiMIQ (**6**) (Table 1 and Figure 8). The second conjugate, **1**, showed cytotoxicity and selectivity similar to that of the reference compound **6** over normal cells (Table 1 and Figure 8).

Drug-induced hemolysis has been identified as a serious side effect of some anticancer therapies and has been proven for some drugs, such as tamoxifen [60], artemisinin derivatives [61], and other bioactive compounds [62]. An additional evaluation was performed using another mammalian cell model, namely freshly isolated human erythrocytes, to gain more information on the possible toxicity of conjugates **1** and **2**. The effect of both conjugates on human red blood cells was tested at a concentration of 1000 nM, which, according to the results presented in Table 1, with one exception (the AsPC-1 data for **1**), exceeded the estimated IC_50_ values for the pancreatic cancer lines. The tested compounds were shown to have negligible hemolytic activity, since the determined hemolysis values were 0.5% and 1.5% for **1** and **2**, respectively. This was in line with our expectations, as even DiMIQ alone caused 50% hemolysis at a much higher concentration, namely 0.12 mM, according to our previously published data [63]. Thus, conjugates **1** and **2** have been shown to be free of harmful hemolytic side effects at a concentration that produces an antitumor activity and have excellent blood compatibility.

### 2.4. Preliminary Synergistic Activity Evaluation

**Figure 9:**
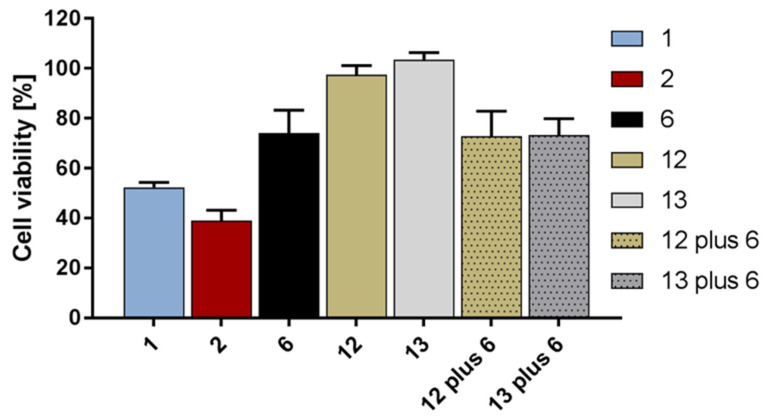
Cytotoxic effects of conjugates **1** and **2**, reference DiMIQ (**6**), HCA derivatives **12** and **13**, **6** plus **12**, **6** plus **13** on BxPC-3 cells after 72 h of incubation at 500 nM concentrations. The results were expressed as the % of viability compared to the untreated control.

One of the expectations for the new conjugates was to increase their cytotoxicity in vitro, compared to the independent effects caused by DiMIQ and hydroxycinnamic acid molecules, or after their co-administration. For this reason, preliminary research was carried out to verify this hypothesis. Thus, compounds **1** and **2**, showing significant activity against BxPC-3, AsPC-1, MCF-7, and HeLa cancer cells, were selected for their evaluation of pseudosynergic action. The pancreatic adenocarcinoma BxPC-3 line was used as a model, and under experimental conditions, a concentration of 500 nM for the compounds was opted for. This was due to the significantly distinguishing cytotoxic effects observed for conjugates **1** and **2** against the DiMIQ reference compound (**6**) under these conditions (Figure 6). The alterations in inhibition of cell growth induced by compounds **1**, **2**, DiMIQ (**6**), methyl para-coumarate (**12**), methyl ortho-coumarate (**13**), and both combinations of **6** with **12** and **13** at 500 nM were analyzed. The **12** and **13** had been selected as simple HCA derivatives previously investigated against pancreatic cancer lines [36]. As shown in Figure 8, compounds **12** and **13** did not influence cancer cells viability. Co-administration of these HCA derivatives together with the highly cytotoxic **6** caused the same cytotoxic response as for **6** administered alone, that is, without the synergism of action (Figure 9). On the contrary, conjugates **1** and **2** showed a significant decrease in cell viability, to approximately 52% and 39%, respectively, compared to approximately 72% induced by DiMIQ (**6**) or the co-administration of **6** with **12** or **13**.

### 2.5. Computational Studies

#### 2.5.1. ADMET Properties

The main predicted ADMET properties of the studied compounds have been presented in Tables S1 and S2. Conjugates **1**–**5** were within the desired common limit of 500 Da, which characterizes systems with good oral bioavailability [64]. They also met Lipinski’s rule of five [65], and the only minor concern from a potential therapeutic point of view may be their logS solubility values, as they were predicted to be below the −5.7 threshold, which does not meet Jorgensen’s rule of three [66]. They were also predicted not to bind strongly to human serum albumin, which would have reduced the amount of drug in the general circulation. Compounds **1**–**3** and DiMIQ (**6**) were also predicted to have very good membrane permeability, while the more hydrophilic compounds **4** and **5** were predicted to be slightly worse, but still within acceptable values for drug-like systems. However, in the context of their potential toxicity, compounds **1**–**5** were predicted to be potential blockers of the HERG K+ channel implicated in fatal arrhythmia [67], although the values obtained were inconclusive. Additional toxicity predictions using ProTox-II [68] showed relatively high LD50 values and no potential toxicity issues, except for the potential immunotoxicity of compounds **1**–**5** (predicted with high confidence) and mutagenicity (predicted with moderate confidence: see Supplementary Materials, Figures S11–S16). Finally, we also predicted cytochrome P450-mediated metabolism for compounds **1**–**5**, using the induced-fit approach. For compounds **1**, **3,** and **4**, the predicted metabolic sites included the phenyl ring atoms of the pyrroloquinoline moiety, as well as the phenol ring atom. However, for compounds **2** and **5**, only the former were predicted as possible metabolic sites, due to the different arrangement of the OH group(s) and likely variation in pose within the cytochrome P450 binding pocket (see Supplementary Materials, Figure S17).

#### 2.5.2. Docking Studies

**Figure 10:**
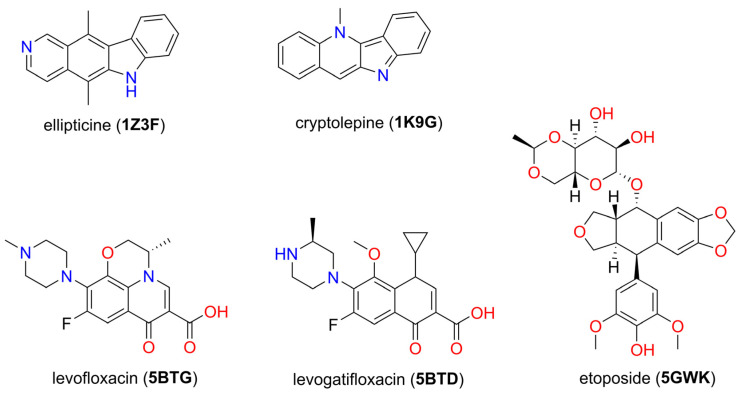
Structures of ligands used in the computational part of this study.

In the molecular docking part of the study, a two-stage approach, with separately performed docking to the DNA model and to topoisomerase II alpha/beta models, was used. This allowed the binding energies of the investigated ligands to be evaluated with both DNA alone and in complex with topoisomerase II, highlighting the differences between these two potential modes of binding. In the first stage of our docking studies, the crystal structure of the DNA complex with ellipticine (PDB code: 1Z3F) was used to perform flexible ligand docking with DNA, treated as a completely rigid system [69]. For the DNA/topoisomerase II docking, four crystal structures of human topoisomerase II alpha (PDB codes: 4FM9, 5GWK, 6ZY5, and 6ZY6) [70,71,72] and four crystal structures of human topoisomerase II beta (PDB codes: 3QX3, 4G0V, 4J3N, and 5GWJ) [73,74,75] were applied. These structures were selected based on the structural variability of the ligand-bound complexes, but also considering the structures of the protein–DNA complexes without any incorporated ligands (4FM9 and 4J3N). We used the same protocol for protein preparation and molecular docking as the one published in our previous work [76]. The predicted Gibbs free energies of binding and the Ki values (Table 2) were the lowest estimates of the molecular docking to four different crystal structures of the receptor. For comparison, we also performed molecular docking of the selected ligands, known for their interaction with DNA/topoisomerase II, that is, ellipticine (PDB code: 1Z3F), cryptolepine (PDB code: 1K9G), 1-cyclopropyl-6-fluoro-8-methoxy-7-[(3S)-3-methylpiperazin-1-yl]-4-oxo-1,4-dihydroquinoline-3-carboxylic acid (PDB code: 5BTD), (3S)-9-fluoro-3-methyl-10-(4-methylpiperazin-1-yl)-7-oxo-2,3-dihydro-7H-[1,4]oxazino [2,3,4-ij]quinoline-6-carboxylic acid (PDB code: 5BTG), and etoposide (PDB code: 5GWK) [69,71,77,78]. The structures of molecules used in the docking studies are shown in Figure 10.

**Figure 11:**
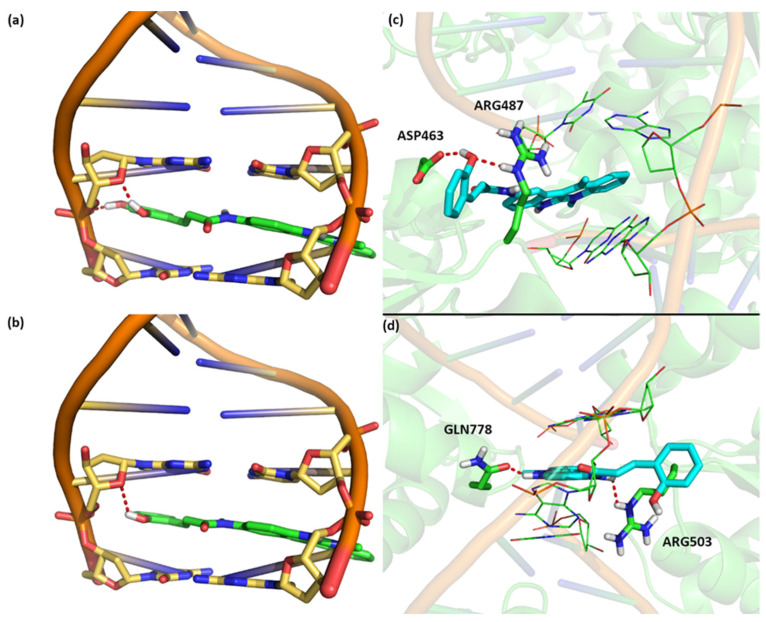
Predicted binding sites and hydrogen bonds between ligands (in green) and DNA (in orange/yellow): (**a**) for compound **4**, (**b**) for compound **2** and its predicted binding sites, as well as the most important interactions in the complex formed by conjugate **2** with DNA, (**c**) for topoisomerase II alpha, and (**d**) for topoisomerase II beta.

The estimated Gibbs free energy of conjugates **1**–**5** was found to be similar, ranging from −8.3 to −9.3 kcal/mol, and approximately 1 kcal/mol stronger (one order of magnitude lower in the Ki value) than the predicted value for DiMIQ (**6**) (see Table 2). The strongest predicted DNA binding for conjugate **4** was due to two hydrogen bonds formed between the hydroxyl groups of the benzene-1,2-diol moiety and the oxygen atom of deoxyribose, as well as the DNA phosphate group. Other derivatives formed only one hydrogen bond with DNA, resulting in a relatively weaker binding property (Figure 11).

Molecular docking of the studied ligands to the topoisomerase II-DNA complexes showed a number of interesting characteristics (Figure 11). First, the binding energy of DiMIQ (**6**) was similar for both the DNA–topoisomerase II complex and the DNA model, indicating that this ligand did not interact with the amino acid residues of the enzyme. On the contrary, conjugates **1**–**5** showed several interactions with the amino acid residues of topoisomerase II, replacing those with parts of the DNA, and improving their binding to the protein–DNA complex, with respect to the DNA model. The highest Gibbs free energy of binding (Gbind) with both topoisomerase II isoforms was found for conjugate **2**. The analysis of the predicted binding site of **2** in the topoisomerase II alpha–DNA complex revealed two strong hydrogen bonds formed by the phenol moiety of the ligand with ASP463 and ARG487, further stabilizing the ligand–DNA interaction (see Figure 11). On the other hand, for topoisomerase II beta, compound **2** also formed two hydrogen bonds: one between the indole moiety and GLN778, and the second one between the amide group and ARG503 (see Figure 11). Given the expected accuracy of the G_bind_ estimation in the molecular docking approach of around 1–2 kcal/mol and the calculated differences in G_bind_ between compounds **1**–**5**, one cannot undoubtedly choose the best candidate for further tests. However, for all the conjugates investigated, the predicted Ki values were two orders of magnitude higher than those of DiMIQ (**6**) and one order of magnitude lower than those for etoposide, suggesting strong binding and a possible high potential as candidates for therapeutic agents (Table 3).

## 3. Materials and Methods

### 3.1. General Information

All reagents and solvents were purchased from common commercial suppliers, without further purification. For monitoring the progress of the reactions, Merck DC-Alufolien Kieselgel 60 F_254_ TLC plates (Merck, Darmstadt, Germany) were used. Column chromatography was performed on Merck silica gel 60, 230–400 mesh (Merck, Darmstadt, Germany). Melting points were measured on a Mettler Toledo MP70 apparatus (Metler Toledo, Greifensee, Switzerland) and were uncorrected. NMR spectra were recorded on a Bruker AVANCE III HD 500 MHz spectrometer (Bruker, Billerica, MA, USA) at 298 K in CDCl_3_ (Merck, Darmstadt, Germany)or DMSO-d6 (Merck, Darmstadt, Germany) using TMS as internal standard (Supplementary Materials, Figures S1–S10). The final structural analysis of the results from 1D and 2D NMR experiments was performed. Two-dimensional spectra were also obtained for compounds in which it was not possible to obtain a clear ^13^C spectrum (for example, due to signal broadening). High-resolution mass spectrometry (HRMS) measurements were performed using a Synapt G2-Si mass spectrometer (Waters Corp., Milford, MA, USA) equipped with an ESI source and quadrupole-time-of-flight mass analyzer. The mass spectrometer operated in positive or negative ion detection modes. Measurement was performed with the capillary voltage set to 2.7 kV, and the sampling cone was set to 20 V. The source temperature was 110 °C. To ensure accurate mass measurements, the data were collected in centroid mode, and the mass was corrected during acquisition using a leucine encephalin solution as external reference, Lock-SprayTM, (Waters Corp., Milford, MA, USA), which generated a reference ion at *m*/*z* 554.2615 ([M−H]^−^) in negative ESI mode and at *m*/*z* 556.2771 Da ([M+H]^+^) in positive ESI mode. The results of the measurements were processed using MassLynx 4.1 software (Waters Corp., Milford, MA, USA). The analysis of the percentage of C, H, N content was performed in an automatic UNIcube analyzer by Elementar company. The Cl content was determined by potentiometric-argentometric titration with AgNO_3_.

MTT (3-(4,5-dimethylthiazol-2-yl)-2,5-diphenyltetrazolium bromide) was purchased from Sigma-Aldrich (Poznan, Poland) and DMSO from Archem (Kamieniec Wroclawski, Poland). Cell culture media (RPMI-1640, alpha-MEM, and DMEM), stable glutamine 100×, Trypsin-EDTA, heat inactivated fetal bovine serum premium (FBS), and antibiotic–antimycotic 100× were purchased from BioWest (BioWest by CytoGen, Zgierz, Poland). The normal human dermal fibroblast cell line (NHDF) was purchased from Lonza (Lonza, Warsaw, Poland), and the BxPC-3 cell line was purchased from the American Tissue Culture Collection (ATCC, Manassas, VA, USA). AsPC-1, MCF 7, and HeLa cell lines were kindly provided by the Institute of Immunology and Experimental Therapy (Wroclaw, Poland). The hydroxycinamic acids (HCAs), chemical reagents, and solvents were purchased from common chemical suppliers. Acetyl-protected HCA **19**–**22** and HCA methyl esters **12**–**15** were synthesized according to the common procedures [50,51]. Compounds **19**–**22** were converted to the corresponding acid chlorides **23**–**26** with an excess of thionyl chloride. 9-amino-DIMIQ was obtained previously from the Pharmaceutical Research Institute (Warsaw, Poland), via a classic modification of the Graebe–Ullmann method used in the synthesis of the indolo[2,3-*b*]quinoline system [43,44].

### 3.2. Chemical Syntesis

#### 3.2.1. General Procedure of Synthesis of Compounds **16**–**18**

Methyl esters **13**–**15** [51] (1 eq.) were dissolved in acetone (30 mL), then K_2_CO_3_ (4 eq.) and allyl bromide (4 eq.) were added. The mixture was stirred overnight at ambient temperature. The inorganic salts were filtered off, and solvents were evaporated to an oily residue. Then methanol (10 mL) and NaOH aq (2 eq., 5 mL) were added and the reaction mixture was stirred at room temperature for 1 h (TLC control). After the reaction had been completed, the mixture was diluted with water (100 mL) and washed with DCM (2 *×* 20 mL). The aqueous layer was adjusted to pH 1.0 with conc. HCl aq, then the product was extracted with DCM (3 *×* 30 mL). The combined organic layers were dried under anhydrous MgSO_4_, then filtered and evaporated to dryness to give allyl protected compounds **16**–**18**.

##### (2-Allyloxy)cinnamic Acid (**16**)

Compound **16** [79] was obtained as a white solid from **13** [51] (1000 mg, 5.62 mM). The yield was 1043 mg (91%); m.p. 159.9 °C (121–123 °C [80]); ^1^H NMR (500 MHz, DMSO-d6) δ 12.31 (bs, 1H), 7.86 (d, 1H, J = 16.2 Hz), 7.68 (dd, 1H, J_1_ = 7.8 Hz, J_2_ = 1.6 Hz), 7.37 (ddd, 1H, J_1_ = J_2_ = 7.8, J_3_ = 1.6 Hz), 7.07 (d, 1H, J = 7.8 Hz), 6.97 (dd, 1H, J_1_ = J_2_ = 7.8), 6.52 (d, 1H, J = 16.2 Hz), 6.08 (ddt, 1H, J_1_ = 17.2 Hz, J_2_ = 10.5 Hz, J_3_ = 5.2 Hz), 5.41 (dd, 1H, J_1_ = 17.2 Hz, J_2_ = 1.6 Hz), 5.29 (dd, 1H, J_1_ = 10.5 Hz, J_2_ = 1.6 Hz), 4.66 (d, 1H, J = 5.2 Hz). ^13^C NMR (125 MHz, DMSO-d6) δ 167.8, 156.6, 138.6, 133.4, 131.6, 128.5, 122.7, 120.9, 119.3, 117.7, 112.9, 68.6, HRMS (ES) *m*/*z*: [M+H]^+^ calc. for C_12_H_13_O_3_: 205.0865, found: 205.0857; [M+Na]^+^: calc. for C_12_H_12_O_3_Na: 227.0684, found: 227.0674; [M−H]^+^ calc. for C_12_H_11_O_3_: 203.0708, found: 203.0707.

##### (2,4-Diallyloxy)cinnamic Acid (**17**)

Compound **17** [54] was obtained as a white solid from **14** [51] (1000 mg, 5.150 mM). The yield was 920 mg (69%); m.p. 149.1 °C; ^1^H NMR (500 MHz, DMSO-d6) δ 12.12 (bs, 1H), 7.77 (d, 1H, J = 16.1 Hz), 7.60 (d, 1H, J = 8.6 Hz), 6.63 (d, 1H, J = 2.3 Hz), 6.58 (dd, 1H, J_1_ = 8.6 Hz, J_2_ = 2.3 Hz), 6.39 (d, 1H, J = 16.1 Hz), 5.98–6.12 (m, 2H), 5.36–5.45 (m, 2H), 5.22–5.33 (m, 2H), 4.57–4.68 (m, 4H). ^13^C NMR (125 MHz, DMSO-d6) δ 168.2, 161.2, 158.1, 138.7, 133.3, 129.9, 117.8, 116.5, 115.7, 107.0, 100.1, 68.8, 68.5, HRMS (ES) *m*/*z*: [M+Na]^+^ calc. for C_15_H_16_O_4_Na: 283.0946, found: 283.0940; [M−H]^+^ calc. for C_15_H_15_O_4_: 259.0970, found: 259.0971.

##### (3,4-Diallyloxy)cinnamic Acid (**18**)

Compound **18** [80] was obtained as a white solid from **15** [51] (1000 mg, 5.150 mM). The yield was 1190 mg (89%); m.p. 161.7 °C (159–160 °C [80]); ^1^H NMR (500 MHz, CDCl_3_) δ 7.70 (d, 1H, J = 15.8 Hz), 7.08–7.13 (m, 2H), 6.87 (d, 1H, J = 8.1 Hz), 6.28 (d, 1H, J = 15.8 Hz), 6.02–6.13 (m, 2H), 5.40–5.47 (m, 2H), 5.28–5.34 (m, 2H), 4.62–4.68 (m, 4H). ^13^C NMR (125 MHz, CDCl_3_) δ 172.6, 151.0, 148.6, 146.9, 133.0, 132.0, 127.2, 123.1, 118.0, 117.9, 114.9, 113.3, 112.8, 69.9, 69.7, HRMS (ES) *m*/*z*: [M+H]^+^ calc. for C_15_H_17_O_4_: 261.1127, found: 261.1139; [M+Na]^+^ calc. for C_15_H_16_O_4_Na: 283.0946, found: 283.0951.

#### 3.2.2. General Procedure for Synthesis of Compounds **27**–**29**

To a solution of allyl-protected HCA **16**–**18** (1.2 eq.), TBTU (1.2 eq.) in DMF (4 mL) and DIPEA (3 eq.) were added. The mixture was stirred for 15 min at room temperature. Then, the solution of 5,11-dimethyl-5*H*-indolo[2,3-*b*]quinolin-9-ylamine (1 eq.) in 2 mL of DMF was added, and the stirring was continued for 6–24 h (TLC monitoring). The crude product was precipitated with water, filtered off, washed with water, and dried under reduced pressure. The crude product was purified through a silica gel column eluted with dichloromethane/methanol (*v*/*v*).

##### 9-[((2-Allyloxy)cinnamoyl)amino]-5,11-dimethyl-5*H*-indolo[2,3-*b*]quinoline (**27**)

Compound **27** was obtained as an orange solid from **16** (188 mg, 0.915 mM) and 5,11-dimethyl-5*H*-indolo[2,3-*b*]quinolin-9-ylamine (200 mg, 0.766 mM). The crude product was purified by chromatography on a silica gel column with dichloromethane–methanol 15:1→1:1 (*v*/*v*). The yield was 234 mg (68%); m.p. 196–198 °C; ^1^H NMR (500 MHz, DMSO-d6) δ 10.30 (s, 1H, NH), 8.77 (d, 1H, J = 1.7 Hz), 8.30 (d, 1H, J = 7.4 Hz), 7.95 (d, 1H, J = 8.6 Hz), 7.93 (d, 1H, J = 15.8 Hz), 7.85 (t, 1H, J = 7.1 Hz), 7.70 (dd, 1H, J_1_ = 8.5 Hz, J_2_ = 1.9 Hz), 7.61 (dd, 1H, J_1_ = 7.6 Hz, J_2_ = 1.3 Hz), 7.48–7.56 (m, 2H), 7.34–7.39 (m, 1H), 7.10 (d, 1H, J = 8.3 Hz), 7.02 (t, 1H, J = 7.5 Hz), 6.90 (d, 1H, J = 15.8 Hz), 6.09–6.19 (m, 1H), 5.42–5.49 (m, 1H), 5.30–5.35 (m, 1H), 4.67–4.71 (m, 2H), 4.24 (s, 3H), 3.09 (s, 3H); 13C NMR (125 MHz, DMSO-d6) δ 163.5 (C=O), 156.4, 136.2, 134.2, 133.5, 132.1, 130.9, 130.8, 127.4, 126.0, 123.8, 123.5, 122.7, 122.0, 120.9, 120.6, 120.2, 117.6 (CH_2_), 116.2, 115.2, 114.4, 113.0, 68.7 (CH_2_), 33.0 (CH_3_), 15.0 (CH_3_); HRMS (ES+) *m*/*z*: [M+H]^+^ calc. for C_29_H_26_N_3_O_2_: 448.2025, found: 448.2025.

##### 9-[((2,4-Diallyloxy)cinnamoyl)amino]-5,11-dimethyl-5*H*-indolo[2,3-*b*]quinoline (**28**)

Compound **28** was obtained as an orange solid from **17** (240 mg, 0.92 mM) and 5,11-dimethyl-5*H*-indolo[2,3-*b*]quinolin-9-ylamine (200 mg, 0.766 mM). The crude product was purified by chromatography on a silica gel column with dichloromethane–methanol 20:1→2:1 (*v*/*v*). The yield was 277 mg (72%); m.p. 220–222 °C; ^1^H NMR (500 MHz, DMSO-d6) δ 10.17 (s, 1H, NH), 8.77 (br, 1H), 8.34 (d, 1H, J = 8.0 Hz), 7.97 (d, 1H, J = 8.6 Hz), 7.87 (t, 1H, J = 7.6 Hz), 7.81 (d, 1H, J = 15.7 Hz), 7.70 (d, 1H, J = 8.2 Hz), 7.48–7.59 (m, 3H), 6.77 (d, 1H, J = 15.7 Hz), 6.60–6.68 (m, 2H), 6.00–6.19 (m, 2H), 5.37–5.50 (m, 2H), 5.23–5.37 (m, 2H), 4.66–4.74 (m, 2H), 4.58–4.64 (m, 2H), 4.25 (s, 3H), 3.11 (s, 3H); ^13^C NMR (125 MHz, DMSO-d6) δ 163.9 (C=O), 160.7, 157.7, 136.1, 134.2, 133.5, 133.4, 132.6, 131.0, 128.7, 126.1, 123.5, 122.3, 120.8, 120.2, 120.0, 117.7 (CH_2_), 116.6, 115.9, 115.3, 114.4, 107.0, 100.2, 68.8 (CH_2_), 68.4 (CH_2_), 33.2 (CH_3_), 15.0 (CH_3_); HRMS (ES+) *m*/*z*: [M+H]^+^ calc. for C_32_H_30_N_3_O_3_: 504.2287, found: 504.2286.

##### 9-[((3,4-Diallyloxy)cinnamoyl)amino]-5,11-dimethyl-5*H*-indolo[2,3-*b*]quinoline (**29**)

Compound **29** was obtained as an orange solid from **18** (180 mg, 0.69 mM) and 5,11-dimethyl-5*H*-indolo[2,3-*b*]quinolin-9-ylamine (150 mg, 0.575 mM). The crude product was purified by chromatography on a silica gel column with dichloromethane–methanol 15:1→1:1 (*v*/*v*). The yield was 217 mg (75%); m.p. 213–215 °C; ^1^H NMR (500 MHz, DMSO-d6) δ 10.16 (s, 1H, NH), 8.76 (d, 1H, J = 1.4 Hz), 8.33 (d, 1H, J = 7.7 Hz), 7.96 (d, 1H, J = 8.6 Hz), 7.82–7.89 (m, 1H), 7.71 (dd, 1H, J_1_ = 8.5 Hz, J_2_ = 1.7 Hz), 7.49–7.56 (m, 3H), 7.24 (d, 1H, J = 1.4 Hz), 7.16 (dd, 1H, J_1_ = 8.3 Hz, J_2_ = 1.5 Hz), 7.04 (d, 1H, J = 8.5 Hz), 6.74 (d, 1H, J = 15.6 Hz), 6.01–6.14 (m, 2H), 5.37–5.48 (m, 2H), 5.24–5.32 (m, 2H), 4.57–4.67 (m, 4H), 4.26 (s, 3H), 3.12 (s, 3H); ^13^C NMR (125 MHz, DMSO-d6) δ 163.4 (C=O), 149.3, 147.9, 139.4, 136.4, 133.7, 133.5, 131.9, 130.7, 127.8, 126.0, 124.1, 121.7, 120.5, 120.4, 120.1, 117.4 (CH_2_), 116.5, 115.1, 114.4, 113.6, 112.1, 68.9 (CH_2_), 68.8 (CH_2_), 48.5, 32.7 (CH_3_), 14.9 (CH_3_); HRMS (ES+) *m*/*z*: [M+H]^+^ calc. for C_32_H_30_N_3_O_3_: 504.2287, found: 504.2283.

#### 3.2.3. General Procedure for Synthesis of Compounds **31**–**34**

5,11-dimethyl-5*H*-indolo[2,3-*b*]quinolin-9-yl-amine (1 eq.) was dissolved in DCM (8 mL) and dry pyridine (1 eq.) and cooled to −20 °C. To the stirred solution on an ice-salt bath, the chlorides **23**–**26** were added portionwise over a period of 15 min., with the temperature kept below −5 °C. The reaction was stirred in the ambient temperature for 4–24 h. After the completion of the reaction, SiO_2_ was added to the mixture, followed by its evaporation to dryness. Then, the crude product was purified using flash chromatography on silica gel with chloroform/methanol.

##### 9-[((4-Acetoxy)cinnamoyl)amino]-5,11-dimethyl-5*H*-indolo[2,3-*b*]quinoline (**31**)

Compound **31** was obtained as a yellow solid from **23** (43 mg, 0.19 mM) and 5,11-dimethyl-5*H*-indolo[2,3-*b*]quinolin-9-ylamine (50 mg, 0.19 mM). The crude product **31** was purified by chromatography on a silica gel column with chloroform–methanol 10:1→5:1 (*v*/*v*). The yield was 30 mg (35%); m.p. 238–240 °C (decomp.); ^1^H NMR (500 MHz, DMSO-d6) δ 10.30 (s, 1H, NH), 8.79 (s, 1H), 8.37 (d, 1H, J = 7.9 Hz), 8.02 (d, 1H, J = 8.4 Hz), 7.91 (t, 1H, J = 7.6 Hz), 7.74 (d, 1H, J = 8.3 Hz), 7.68 (d, 2H, J = 8.4 Hz), 7.61 (d, 1H, J = 15.7 Hz), 7.53–7.59 (m, 2H), 7.22 (d, 2H, J = 8.4 Hz), 6.85 (d, 1H, J = 15.7 Hz), 4.29 (s, 3H), 3.15 (s, 3H), 2.29 (s, 3H, CH_3_); ^13^C NMR (125 MHz, DMSO-d6) δ 163.1 (C=O), 138.6 (C-4′), 132.2, 130.0, 128.8 (C-2″), 122.7 (C-3′), 122.5 C-3″), 122.3, 115.4, 126.2, 120.4 (C-8), 116.2, 114.6 (C-10), 33.2 (CH_3_), 20.9 (CH_3_-Ac), 15.1 (CH_3_); HRMS (ES+) *m*/*z*: [M+H]^+^ calc. for C_28_H_24_N_3_O_3_: 450.1818, found: 450.1810.

##### 9-[((3-Acetoxy)cinnamoyl)amino]-5,11-dimethyl-5*H*-indolo[2,3-*b*]quinoline (**32**)

Compound **32** was obtained as a yellow solid from **24** (129 mg, 0.574 mM) and 5,11-dimethyl-5*H*-indolo[2,3-*b*]quinolin-9-ylamine (150 mg, 0.574 mM). The crude **32** was purified by chromatography on a silica gel column with chloroform–methanol 10:1→5:1 (*v*/*v*). The yield was 94 mg (36%); m.p. 235–237 °C (decomp.); ^1^H NMR (500 MHz, DMSO-d6) δ 10.56 (s, 1H, NH), 8.95 (br, 1H), 8.60 (br, 1H), 8.30 (br, 1H), 8.09 (br, 1H), 7.83–7.90 (m, 1H), 7.79 (br, 1H), 7.70 (d, 1H, J = 8.3 Hz), 7.63 (d, 1H, J = 15.6 Hz), 7.47–7.57 (m, 2H), 7.41 (s, 1H), 7.20 (d, 1H, J = 7.3 Hz), 6.91 (d, 1H, J = 15.6 Hz), 4.41 (s, 3H), 3.13 (s, 3H), 2.31 (s, 3H, CH_3_); ^13^C NMR (125 MHz, DMSO-d6) δ 169.2 (CO-Ac), 163.2 (C=O), 151.0, 139.0 (C-4′), 136.4, 130.2, 126.8, 125.2, 123.4 (C-3′), 123.2, 120.8 (C-2), 114.5 (C-10), 20.9 (CH_3_-Ac), 15.7 (CH_3_); HRMS (ES+) *m*/*z*: [M+H]^+^ calc. for C_28_H_24_N_3_O_3_: 450.1818, found: 450.1816.

##### 9-[((2,4-Diacetoxy)cinnamoyl)amino]-5,11-dimethyl-5*H*-indolo[2,3-*b*]quinoline (**33**)

Compound **33** was obtained as a yellow solid from **25** (162 mg, 0.574 mM) and 5,11-dimethyl-5*H*-indolo[2,3-*b*]quinolin-9-ylamine (150 mg, 0.574 mM). The crude product **33** was purified by chromatography on a silica gel column with chloroform–methanol 10:1→5:1 (*v*/*v*). The yield was 172 mg (58%); m.p. 245–247 °C (decomp.); ^1^H NMR (500 MHz, DMSO-d6) δ 10.53 (s, 1H, NH), 8.87 (s, 1H), 8.44 (d, 1H, J = 7.6 Hz), 8.11 (d, 1H, J = 7.3 Hz), 7.95 (t, 1H, J = 7.3 Hz), 7.72–7.82 (m, 2H), 7.57–7.68 (m, 2H), 7.55 (d, 1H, J = 15.7 Hz), 7.19 (d, 1H, J = 8.3 Hz), 7.14 (br, 1H), 6.95 (d, 1H, J = 15.7 Hz), 4.33 (s, 3H), 3.19 (s, 3H), 2.41 (s, 3H, CH_3_), 2.29 (s, 3H, CH_3_); ^13^C NMR (125 MHz, DMSO-d6) δ 169.0 (CO-Ac), 168.9 (CO-Ac), 151.7, 149.4, 133.2, 132.8 (C-4′), 128.3 (C-6″), 126.9, 124.3 (C-3′), 121.3, 120.4 (C-5″), 117.3 (C-3″), 116.9, 114.7 (C-10), 36.4 (CH_3_), 20.8 (CH_3_-Ac), 20.7 (CH_3_-Ac), 15.9 (CH_3_); HR-MS (ES+) *m*/*z*: [M+H]^+^ calc. for C_30_H_26_N_3_O_5_: 508.1872, found: 508.1868.

##### 9-[((3,4-Diacetoxy)cinnamoyl)amino]-5,11-dimethyl-5*H*-indolo[2,3-*b*]quinoline (**34**)

Compound **34** was obtained as a yellow solid from **26** (100 mg, 0.383 mM) and 5,11-dimethyl-5*H*-indolo[2,3-*b*]quinolin-9-ylamine (100 mg, 0.383 mM). The crude product **34** was purified by chromatography on a silica gel column with chloroform–methanol 10:1→5:1 (*v*/*v*). The yield was 88 mg (45%); m.p. 273–275 °C (decomp.); ^1^H NMR (500 MHz, DMSO-d6) δ 10.63 (s, 1H, NH), 8.83 (s, 1H), 8.56 (d, 1H, J = 8.3 Hz), 8.29 (d, 1H, J = 8.7 Hz), 8.04 (t, 1H, J = 7.7 Hz), 7.72–7.80 (m, 2H), 7.48–7.60 (m, 4H), 7.36 (d, 1H, J = 8.2 Hz), 6.80 (d, 1H, J = 15.7 Hz), 4.44 (s, 3H, CH_3_), 3.24 (s, 3H, CH_3_), 2.33 (s, 3H, CH_3_), 2.31 (s, 3H, CH_3_); ^13^C NMR (125 MHz, DMSO-d6) δ 168.2, 168.1, 163.2, 142.8, 142.3, 138.2, 135.3, 134.9, 133.5, 133.0, 126.8, 125.9, 125.3, 124.2, 123.2, 122.7, 122.5, 120.6, 120.5, 116.8, 114.0, 112.7, 36.5, 20.3, 15.8; HRMS (ES+) *m*/*z*: [M+H]^+^ calc. for C_30_H_26_N_3_O_5_: 508.1872, found: 508.1872.

##### 9-[((2-Hydroxy)cinnamoyl)amino]-5,11-dimethyl-5*H*-indolo[2,3-*b*]quinoline (**2**) Dihydrochloride

The compound **27** (144 mg,0.32 mM), triethylsilane (51 µL,0.32 mM), and tetrakis(triphenylphosphine)palladium (0) (185 mg, 0.16 mM) were dissolved in DMF (6 mL) in a dark flask, protected from light. The reaction mixture was stirred for 5 h. After the completion of the reaction, the solvent was evaporated under reduced pressure at ca. 40 °C. The resulting oil was diluted in MeOH and the SiO_2_ (2 g) was added to the mixture, followed by its evaporation to dryness. The loaded-on silica gel crude product was then purified by silica gel chromatography with chloroform–methanol 20:1→2:1 (*v*/*v*) as an eluent system to afford compound **2** as a red solid. The yield was 64 mg (50%); m.p. 265–267 °C (decomp.); ^1^H NMR (500 MHz, DMSO-d6) δ 10.12–10.30 (m, 2H, OH, NH-1′), 8.79 (d, 1H, H-10, J = 1.4 Hz), 8.32 (d, 1H, H-1, J = 8.2 Hz), 7.95 (d, 1H, H-4, J = 8.5 Hz), 7.82–7.87 (m, 1H, H-3), 7.80 (d, 1H, H-4′, J = 15.8 Hz), 7.74 (dd, 1H, H-8, J_1_ = 8.5 Hz, J_2_ = 1.8 Hz), 7.53 (d, 1H, H-7, J = 8.5 Hz), 7.49–7.54 (m, 1H, H-2), 7.46–7.52 (m, 1H, H-6″), 7.18–7.23 (m, 1H, H-4″), 6.96 (d, 1H, H-3′, J = 15.7 Hz), 6.94 (d, 1H, H-3″, J = 7.7 Hz), 6.86 (t, 1H, H-5″, J = 7.4 Hz), 4.26 (s, 3H, H-5b), 3.12 (s, 3H, H-11b); ^13^C NMR (125 MHz, DMSO-d6) δ 163.8 (C=O, C-2′), 156.5 (C-2″), 154.6 (C-5a), 151.1 (C-6a), 140.4 (C-11), 136.4 (C-4a), 135.5 (C-4′), 131.9 (C-9), 130.6 (C-3), 130.6 (C-4″), 128.6 (C-6″), 126.0 (C-1), 124.5 (C-10b), 124.3 (C-10a), 122.2 (C-3′), 121.7 (C-1″), 121.5 (C-2), 120.4 (C-11a), 120.2 (C-8), 119.4 (C-5″), 116.8 (C-7), 116.2 (C-3″), 115.0 (C-4), 114.5 (C-10), 32.6 (C-5b), 14.9 (C-11b); HRMS (ES+) *m*/*z*: [M+H]^+^ calc. for C_26_H_22_N_3_O_2_: 408.1712, found: 408.1711. Compound **2** was converted into its respective dihydrochloride by using 2M HClg/AcOEt. ^1^H NMR (500 MHz, DMSO-d6) δ 13.96 (s, 1H. NH+-6), 10.57 (s, 1H, NH-1′), 10.24 (s, 1H, OH), 9.01 (s, 1H, H-10), 8.65 (d, 1H, H-1, J = 7.8 Hz), 8.36 (d, 1H, H-4, J = 7.9 Hz), 8.10–8.17 (m, 1H, H-3), 7.95 (d, 1H, H-8, J = 8.1 Hz), 7.81–7.89 (m, 1H, H-2), 7.79–7.86 (m, 1H, H-4′), 7.73 (d, 1H, H-7, J = 8.6 Hz), 7.49 (d, 1H, H-6″, J = 7.1 Hz), 7.22 (d, 1H, H-4″, J = 6.9 Hz), 6.96–7.02 (m, 1H, H-3′), 6.94–6.99 (m, 1H, H-3″), 6.84–6.90 (m, 1H, H-5″), 4.47 (s, 3H, H-5b), 3.33 (s, 3H, H-11b); anal. calc. for C_26_H_21_N_3_O_3_ × 2HCl × 2H_2_O [515.14]: C 60.47, H 5.27, N 8.14, Cl 13.73; found: C 60.39, H 5.48, N 8.47, Cl 13.22.

##### 9-[(2,4-Dihydroxy)cinnamoyl)amino]-5,11-dimethyl-5*H*-indolo[2,3-*b*]quinoline (**4**) Dihydrochloride

Compound **28** (105 mg, 0.21 mM), triethylsilane (34 µL, 0.21 mM), and tetrakis(triphenylphosphine)palladium (0) (241 mg, 0.21 mM) were dissolved in DMF (5 mL) in a dark flask, protected from light. The reaction mixture was stirred for 1 h. After the completion of the reaction, the solvent was evaporated under reduced pressure at ca. 40 °C. The resulting oil was diluted in MeOH and the SiO_2_ (1.5 g) was added to the mixture, followed by its evaporation to dryness. The loaded-on silica gel crude product was then purified by silica gel chromatography with chloroform–methanol 20:1→2:1 (*v*/*v*) as an eluent system to afford compound **4** as a red solid. the yield was 30 mg (34%); m.p. 258–260 °C (decomp.). ^1^H NMR (500 MHz, DMSO-d6) δ 10.10 (s, 2H, NH-1′, OH), 9.81 (brs, 1H, OH), 8.80 (d, 1H, H-10, J = 1.3 Hz), 8.33 (d, 1H, H-1, J = 7.5 Hz), 7.96 (d, 1H, H-4, J = 7.5 Hz), 7.82–7.88 (m, 1H, H-3), 7.73 (dd, 1H, H-8, J_1_ = 8.6 Hz, J_2_ = 1.7 Hz), 7.70 (d, 1H, H-4′, J = 15.7 Hz), 7.49–7.54 (m, 1H, H-2), 7.48–7.53 (m, 1H, H-7), 7.29 (d, 1H, H-6″, J = 8.5 Hz), 6.76 (d, 1H, H-3′, J = 15.7 Hz), 6.42 (d, 1H, H-3″, J = 2.2 Hz), 6.31 (dd, 1H, H-5″, J_1_ = 8.4 Hz, J_2_ = 2.2 Hz), 4.27 (s, 3H, H-5b), 3.13 (s, 3H, H-11b); ^13^C NMR (125 MHz, DMSO-d6) δ 164.5 (C-2′), 160.0 (C-4″), 158.0 (C-2″), 154.5 (C-5a), 150.9 (C-6a), 140.3 (C-11), 136.5 (C-4a), 135.9 (C-4′), 132.2 (C-9), 130.6 (C-3), 129.9 (C-6″), 126.0 (C-1), 124.6 (C-10b), 124.3 (C-10a), 121.5 (C-2), 120.4 (C-11a), 120.1 (C-8), 118.2 (C-3′), 116.7 (C-7), 115.0 (C-4), 114.4 (C-10), 113.4 (C-1″), 107.5 (C-5″), 102.7 (C-3″), 32.6 (C-5b), 14.9 (C-11b); HRMS (ES+) *m*/*z*: [M+H]^+^ calc. for C_26_H_22_N_3_O_3_: 424.1661, found: 424.1660. Compound **4** was converted into its respective dihydrochloride by using 2M HClg/AcOEt. ^1^H NMR (500 MHz, DMSO-d6) δ 13.96 (s, 1H. NH+−6), 10.41 (s, 1H, NH-1′), 9.81 (s, 1H, OH), 9.01 (s, 1H, H-10), 8.66 (d, 1H, H-1, J = 8.2 Hz), 8.37 (d, 1H, H-4, J = 8.8 Hz), 8.11–8.17 (m, 1H, H-3), 7.93 (d, 1H, H-8, J = 8.6 Hz), 7.83–7.89 (m, 1H, H-2), 7.70–7.76 (m, 1H, H-4′), 7.70–7.74 (m, 1H, H-7), 7.30 (d, 1H, H-6″, J = 8.5 Hz), 6.77 (d, 1H, H-3′, J = 15.6 Hz), 6.42 (d, 1H, H-3″, J = 2.2 Hz), 6.31 (dd, 1H, H-5″ J_1_ = 8.5 Hz, J_2_ = 2.2 Hz’), 4.47 (s, 3H, H-5b), 3.33 (s, 3H, H-11b); anal. calc. for C_26_H_21_N_3_O_3_ × 2HCl × H_2_O [513.12]: C 60.71, H 4.90, N 8.17, Cl 13.78; found: C 60.65, H 4.80, N 8.58, Cl 13.51.

##### 9-[((4-Hydroxy)cinnamoyl)amino]-5,11-dimethyl-5*H*-indolo[2,3-*b*]quinoline (**1**) Dihydrochloride

Compound **31** (20 mg, 0.044 mM) was dissolved in DMF (1 mL), and K_2_CO_3_ (7.4 mg, 0.053 mM) was added. The red mixture was stirred for 2 h at room temperature. Then, the solvent was evaporated, and the residue was purified by chromatography on a silica gel column with dichloromethane–methanol 20:1→1:1 (*v*/*v*) to afford compound **1** as an orange solid. The yield was 14 mg (78%); m.p. 240–242 °C (decomp.); ^1^H NMR (500 MHz, DMSO-d6) δ 10.07 (s, 1H, NH-1′), 8.77 (s, 1H, H-10), 8.32 (d, 1H, H-1, J = 7.3 Hz), 7.94 (d, 1H, H-4, J = 8.0 Hz), 7.80–7.89 (m, 1H, H-3), 7.67–7.74 (m, 1H, H-8), 7.49–7.56 (m, 1H, H-2), 7.47–7.53 (m, 1H, H-7), 7.41 (d, 1H, H-4′, J = 15.3 Hz), 7.24 (d, 2H, H-2″, H-6″, J = 7.5 Hz), 6.44–6.51 (m, 2H, H-3″, H-5″), 6.39–6.47 (m, 1H, H-3′), 4.25 (s, 3H, H-5b), 3.12 (s, 3H, H-11b); ^13^C NMR (125 MHz, DMSO-d6) δ 168.3 (C-4″), 164.7 (C=O, C-2′), 154.6 (C-5a), 150.8 (C-6a), 141.2 (C-4′), 140.5 (C-11), 136.5 (C-4a), 132.5 (C-9), 130.7 (C-3), 129.8 (C-2″,C-6″), 126.1 (C-1), 124.7 (C-10b), 124.4 (C-10a), 121.7 (C-2), 120.5 (C-11a), 120.2 (C-8), 119.8 (C-1″), 118.0 (C-3″, C-5″), 116.8 (C-7), 115.1 (C-4), 114.5 (C-10), 113.9 (C-3′), 32.7 (C-5b), 15.0 (C-11b); HRMS (ES+) *m*/*z*: [M+H]^+^ calc. for C_26_H_22_N_3_O_2_: 408.1712, found: 408.1715. Compound **1** was converted into its respective dihydrochloride by using 2M HClg/AcOEt. ^1^H NMR (500 MHz, DMSO-d6) δ 14.34 (s, 1H. NH+-6), 10.64 (s, 1H, NH-1′), 10.08 (s, 1H, OH), 8.84 (s, 1H, H-10), 8.54 (d, 1H, H-1, J = 8.1 Hz), 8.27 (d, 1H, H-4, J = 8.7 Hz), 8.00–8.08 (m, 1H, H-3), 7.74–7.82 (m, 1H, H-8), 7.72–7.80 (m, 1H, H-2), 7.55 (d, 1H, H-7, J = 8.7 Hz), 7.44 (d, 2H, H-2″, H-6″, J = 8.3 Hz), 7.47 (d, 1H, H-4′, J = 15.6 Hz), 6.86 (d, 2H, H-3″, H-5″, J = 8.3 Hz), 6.71 (d, 1H, H-3′, J = 15.6 Hz), 4.47 (s, 3H, H-5b), 3.22 (s, 3H, H-11b); anal. calc. for C_26_H_21_N_3_O_2_ × 2HCl × H_2_O [352.25]: C 57.96, H 5.44, N 11.93, Cl 20.13; found: C 58.32, H 5.40, N 12.17, Cl 20.30.

##### 9-[((3-Hydroxy)cinnamoyl)amino]-5,11-dimethyl-5*H*-indolo[2,3-*b*]quinoline (**3**) Dihydrochloride

Compound **32** (60 mg, 0.133 mM) was dissolved in DMF (3 mL), and K_2_CO_3_ (55 mg, 0.4 mM) was added. The red mixture was stirred for 4 h. Then, the solvent was evaporated, and the residue was purified by chromatography on a silica gel column with dichloromethane–methanol 10:1→1:1 (*v*/*v*) to afford compound **3** as an orange solid. The yield was 43 mg (84%); m.p. 263–265 °C (decomp.); ^1^H NMR (500 MHz, DMSO-d6) δ 10.43 (s, H, NH-1′), 9.76 (brs, H, OH,), 8.80 (s, 1H, H-10), 8.34 (d, 1H, H-1, J = 8.2 Hz), 7.97 (d, 1H, H-4, J = 8.5 Hz), 7.83–7.89 (m, 1H, H-3), 7.75 (d, 1H, H-8, J1 = 8.5 Hz), 7.51–7.55 (m, 1H, H-7), 7.46–7.52 (m, 1H, H-2), 7.49 (d, 1H, H-4′, J = 15.9 Hz), 7.21–7.26 (m, 1H, H-5″), 7.02–7.06 (m, 1H, H-6″), 7.00–7.04 (m, 1H, H-2″), 6.88 (d, 1H, H-3′, J = 15.7 Hz), 6.80–6.84 (m, 1H, H-4″), 4.27 (s, 3H, H-5b), 3.14 (s, 3H, H-11b); ^13^C NMR (125 MHz, DMSO-d6) δ 163.2 (C=O, C-2′), 157.8 (C-3″), 154.6 (C-5a), 151.1 (C-6a), 140.6 (C-11), 139.6 (C-4′), 136.5 (C-4a), 136,2 (C-1″), 131.8 (C-9), 130.7 (C-3), 130.0 (C-5″), 126.1 (C-1), 124.5 (C-10b), 124.4 (C-10a), 122.5 (C-3′), 121.6 (C-2), 120.5 (C-11a), 120.2 (C-8), 118.8 (C-6″), 116.9 (C-7), 116.8 (C-4″), 115.1 (C-4), 114.6 (C-10), 113.8 (C-2″), 32.7 (C-5b), 15.0 (C-11b); HRMS (ES+) *m*/*z*: [M+H]^+^ calc. for C_26_H_22_N_3_O_2_: 408.1712, found: 408.1713. Compound **3** was converted into its respective dihydrochloride by using 2M HClg/AcOEt. NMR (500 MHz, DMSO-d6) δ 10.75 (s, 1H, NH-1′), 9.76 (s, 1H, OH’’), 8.88 (s, 1H, H-10), 8.57 (d, 1H, H-1, J = 8.2 Hz), 8.30 (d, 1H, H-4, J = 8.7 Hz), 8.03–8.09 (m, 1H, H-3), 7.80–7.84 (m, 1H, H-8), 7.75–7.82 (m, 1H, H-2), 7.60 (d, 1H, H-7, J = 8.6 Hz), 7.48 (d, 1H, H-4′, J = 15.6 Hz), 7.24 (t, 1H, H-5″, J = 8.0 Hz), 7.00–7.03 (m, 1H, H-6″), 6.99–7.04 (m, 1H, H-2″), 6.82–6.87 (m, 1H, H-3′), 6.81–6.86 (m, 1H, H-4″), 4.47 (s, 3H, H-5b), 3.25 (s, 3H, H-11b); anal. Calc. for C_26_H_21_N_3_O_2_ × 2HCl × 2H_2_O [515.14]: C 60.47, H 5.27, N 8.14, Cl 13.73; found: C 60.59, H 5.23, N 8.24, Cl 13.43.

##### 9-[(3,4-Dihydroxy)cinnamoyl)amino]-5,11-dimethyl-5*H*-indolo[2,3-*b*]quinoline (**5**) Dihydrochloride

Compound **34** (60 mg, 0.118 mM) was dissolved in DMF (3 mL), and K_2_CO_3_ (33 mg, 0.24 mM) was added. The red mixture was stirred for 5 h. Then, the solvent was evaporated, and the residue was purified by chromatography on a silica gel column with dichloromethane–methanol 10:1→1:1 (*v*/*v*) to afford compound **5** as an orange solid. The yield was 40 mg (80%); m.p. 203–205 °C (decomp.); ^1^H NMR (500 MHz, DMSO-d6) δ 10.38 (s, 1H, NH), 9.51 (brs, 1H, OH), 9.30 (brs, 1H, OH), 8.80 (s, 1H, H-10), 8.33 (d, 1H, H-1, J = 8.1 Hz), 7.96 (d, 1H, H-4, J = 8.6 Hz), 7.82–7.88 (m, 1H, H-3), 7.76 (d, 1H, H-8, J = 8.4 Hz), 7.49–7.55 (m, 1H, H-2), 7.49–7.53 (m, 1H, H-7), 7.42 (d, 1H, H-4′, J = 15.5 Hz), 7.06 (s, 1H, H-2″), 6.90 (d, 1H, H-6″, J = 7.5 Hz), 6.81 (d, 1H, H-5″, J = 8.0 Hz), 6.69 (d, 1H, H-3′, J = 15.5 Hz), 4.27 (s, 3H, H-5b), 3.12 (s, 3H, H-11b); ^13^C NMR (125 MHz, DMSO-d6) δ 163.7 (C-2′), 154.3 (C-5a), 150.4 (C-6a), 147.6 (C-4″), 145.6 (C-3″), 140.9 (C-11), 139.9 (C-4′), 136.4 (C-4a), 132.2 (C-9), 130.7 (C-3), 126.4 (C-1″), 126.2 (C-1), 124.3 (C-10b), 124.1 (C-10a), 121.7 (C-2), 120.6 (C-6″), 120.6 (C-11a), 120.2 (C-8), 118.9 (C-3′), 116.6 (C-7), 115.9 (C-5″), 115.1 (C-4), 114.5 (C-10), 114.0 (C-2″), 32.8 (C-5b), 15.0 (C-11b); HRMS (ES+) *m*/*z*: [M+H]^+^ calc. for C_26_H_22_N_3_O_3_: 424.1661, found: 424.1659. Compound **5** was converted into its respective dihydrochloride by using 2M HClg/AcOEt. ^1^H NMR (500 MHz, DMSO-d6) δ 14.33 (s, 1H, NH+-6), 10.65 (s, 1H, NH-1′), 9.54 (s, 1H, OH), 9.33 (s, 1H, OH), 8.91 (s, 1H, H-10), 8.57 (d, 1H, H-1, J = 8.2 Hz), 8.29 (d, 1H, H-4, J = 8.7 Hz), 8.04–8.10 (m, 1H, H-3), 7.80–7.85 (m, 1H, H-8), 7.76–7.82 (m, 1H, H-2), 7.60 (d, 1H, H-7, J = 8.6 Hz), 7.41 (d, 1H, H-4′, J = 15.5 Hz), 7.06 (s, 1H, H-2″), 6.91 (d, 1H, H-6″, J = 8.1 Hz), 6.81 (d, 1H, H-5″, J = 8.1 Hz), 6.65 (d, 1H, H-3′, J = 15.5 Hz), 4.47 (s, 3H, H-5b), 3.25 (s, 3H, H-11b); anal. calc. for C_26_H_21_N_3_O_3_ × 2HCl × 2H_2_O [531.12]: C 58.65, H 5.11, N 7.89, Cl 13.32; found: C 58.80, H 4.85, N 7.64, Cl 12.90.

##### 5,11-Dimethyl-5*H*-indolo[2,3-*b*]quinolin-9-yl-acetamide (**30**)

To the cooled and stirred solution of **22** (121 mg, 0.459 mM) in DMF (4 mL), DCC (103 mg, 0.498 mM) and DMAP (12 mg, 0.096 mM) in DMF (2 mL) were added. The temperature was kept at c.a. 0 °C for 30 min. Then, the solution of 5,11-dimethyl-5*H*-indolo[2,3-*b*]quinolin-9-ylamine (100 mg, 0.383 mM) in 2 mL of DMF was added. The reaction mixture was stirred at the ambient temperature for 7 h. After reaction completion, SiO_2_ (2.0 g) was added to the mixture, followed by its evaporation to dryness. The crude product, loaded onto silica gel, was then purified by silica gel chromatography with dichloromethane–methanol 10:1→3:1 (*v*/*v*) as an eluent system, to afford the migration impurity product **30** as a red solid. The yield was 107 mg (92%); m.p. 200–202 °C (decomp.); ^1^H NMR (500 MHz, DMSO-d6) δ 9.97 (s, 1H, NH), 8.55 (d, 1H, J = 1.9 Hz), 8.30 (d, 1H, J = 7.5 Hz), 7.95 (d, 1H, J = 8.5 Hz), 7.82–7.88 (m, 1H), 7.59 (dd, 1H, J = 8.5 Hz, J = 1.9 Hz), 7.52 (t, 1H, J = 7.5 Hz), 7.47 (d, 1H, J = 8.5 Hz), 4.23 (s, 3H, CH3), 3.05 (s, 3H, CH_3_), 2.08 (s, 3H, CH_3_); ^13^C NMR (125 MHz, DMSO-d6) δ 167.7 (C=O), 141.3, 136.2, 132.1, 130.8, 126.0, 123.7, 121.9, 120.6, 120.2, 116.0, 115.1, 114.4, 32.9 (CH_3_), 23.9 (CH_3_), 14.9 (CH_3_); m.p. 200–202 °C (decomp.); HRMS (ES+) *m*/*z*: [M+H]^+^ calc. for C_19_H_18_N_3_O: 304.1450, found: 304.1453.

### 3.3. Cell Culture

In vitro cell culture procedures were performed under aseptic conditions, and the cells were propagated in a humidified Innova CO-180 incubator (New Brunswick Scientific, Edison, NJ, USA), supplied with 5% CO_2_, and maintained at 37 °C. Subculturing was performed twice per week (at approximately 72 h intervals), and cell growth was monitored with a Nikon Eclipse microscope. Pancreatic cancer cell lines: BxPC-3 (from primary tumor) and AsPC-1 (from ascites) were cultured in a RPMI-1640 medium, supplemented with 10% heat-inactivated fetal bovine serum (FBS), glutamine and an antibiotic–antimycotic mixture. NHDF grew in alpha-MEM. HeLa and MCF-7 were cultivated in DMEM. Both cell culture media were supplemented with 10% FBS, 2 mM glutamine, and antibiotic–antimycotic.

### 3.4. Cell Culture

The cytotoxicity impact of the ligands on cancer cell and healthy control cell lines was evaluated, as described earlier [81,82], by performing the MTT assay [58]. The cells were seeded in 96-well plates (at a density of 5 × 103 cells per well) in an appropriate culture medium, and the plates were incubated for 24 h. Afterwards, the media were replaced with fresh media, supplemented with varying concentrations (in the range of 125–4000 nM) of synthesized complexes (the compounds were dissolved in DMSO, heated, and sonicated at 55 °C for 15 min before dilution in medium) or an equivalent volume of DMSO, considered as solvent control, and the incubation proceeded for another 72 h. Subsequently, the medium containing the chemicals tested was removed, and the MTT solution (50 μL per well of the solution was 10 times diluted in a medium from the solution stock 0.5 mg/mL) was added. After 3 h of incubation, the MTT solution was replaced with DMSO (50 μL/well) to dissolve the purple formazan crystals and developed color. Absorbance was measured at 560 nm with a reference wavelength of 670 nm on an Asys UVM 340 microplate reader (Cambridge, UK). The results were expressed as a percentage of viable cells in comparison to the control (the untreated cells, taken as 100%) by implementing the formula:

Cell Viability (%) = (AT/AC) × 100 where

AT = absorbance of the treated cells, AC = absorbance of the untreated cells.

### 3.5. Determination of Hemolytic Activity

The hemolytic activity was studied using the method described by Jaromin et al. [63] and the procedure approved by the Bioethics Commission at the Lower Silesian Medical Chamber (1/PNHAB/2018). First, conjugates **1** and **2** were dissolved in DMSO and then added in a volume corresponding to a final concentration of 1000 nM. Subsequently, the compounds were thoroughly mixed with a PBS buffer and human erythrocytes and further incubated for 30 min (37 °C). Finally, after centrifugation, the level of released hemoglobin was evaluated spectrophotometrically at a wavelength of 540 nm. Measurements for the appropriate controls—DMSO, positive (erythrocytes in water) and negative (erythrocytes in PBS)—were also performed.

### 3.6. Computational Methods

In the computational part of this work, the models of all studied ligands were built according to our recent work and starting from the structure of the 5,11-dimethyl-5*H*-indolo[2,3-*b*]quinoline (Pubchem CID: 133982) [76]. LigProp 3.3 software (Schrodinger Inc., New York, NY, USA) was used to prepare all-atom 3D structures. Subsequently, we evaluated all ADME properties using QikProp 4.6 software (Schrodinger Inc.) with default options (Table 1). In the molecular docking part, we used the same protocol for protein preparation and molecular docking as in our previous studies [76,83,84]. In short, we used Autodock 4.2 [85] with the Genetic Lamarckian Algorithm and standard options, but including 200 dockings per compound and 5,000,000 energy evaluations per docking [86]. During the docking, the following residues were treated as flexible: ASP463, ARG487, MET766 of chain B for topoisomerase II alpha and ASP 479, ARG503, GLU522, GLN778, and MET782 of chain A for topoisomerase II beta, while all other amino acid residues and DNA were treated as rigid. In each case, we used a 40 × 40 × 40 Å box (30 × 30 × 30 Å box for docking to 1Z3F DNA), centered on the ligand from the crystal structure. For docking to the structures with no ligand (PDB codes: 4FM9 and 4J3N), we first structurally aligned them to crystal structures with ligands (PDB codes: 5GWK and 3QX3, respectively) and then used the same docking boxes as for the latter proteins.

## 4. Conclusions

The five new amide conjugates of natural hydroxycinnamic (ortho-, meta-, para-coumaric, 2,4-dihydroxycinnamic, caffeic acid) and known cytostatic 5,11-dimethyl-5*H*-indolo[2,3-*b*]quinoline (DiMIQ) were synthesized using two different protection-deprotection synthetic strategies. The conjugates of para- and ortho-coumaric acids (**1** and **2**) showed significant, dose-dependent cytotoxic activities against pancreatic cancers BxPC-3 and AsPC-1. Compound **2** exhibited high cytotoxicity against both pancreatic cancer cells with IC_50_ c.a. 400 nM. Particularly interesting results were obtained for the metastatic line, where compound 2 caused a 50% inhibition in a c.a. 5-time lower concentration than the reference DiMIQ (**6**). Conjugates **1**–**3** also displayed high antiproliferative activities towards hormone-dependent breast cancer and cervical cancer cells (MCF-7 and HeLa). Furthermore, the most active conjugate **2** was selective to both types of pancreatic cancer cells with the selectivity index around 5. The potential additive or synergistic effects of DiMIQ (**6**) with HCA derivatives **12**, **13** (all compounds at a 500 nM concentration) were tested in BxPC-3 cells. While co-administering DiMIQ (**6**) with simple HCA derivatives **12**, **13,** no synergistic effect was observed. However, a considerable decrease in cell viability was observed for conjugates **1** and **2**, compared to data collected for **6** alone or with respective methyl coumarates **12** and **13**. This positively verified the hypothesis about the enhanced activity of conjugates, i.e., active molecules linked together via a covalent bond. To estimate the toxic effects of **1** and **2** on blood cells, their hemolytic potential was determined. Both conjugates caused negligible lysis of human erythrocytes, proving that they are blood compatible. The ADMET profiles calculated for compounds **1**–**5** revealed that all conjugates met the Lipinski’s rule of five but did not meet Jorgensen’s rule of three due to their logS solubility values. A molecular docking study revealed several interesting observations. For DNA binding, the estimated Gibbs free energies of all conjugates investigated were similar, i.e., between −8.6 and −9.3 kcal/mol, and around 1–2 kcal/mol higher than for DiMIQ (**6**). Docking studies to topoisomerase II-DNA complexes revealed that DiMIQ (**6**) binding energy was very similar to that of the DNA case, indicating no additional interactions of DiMIQ (**6**) with topoisomerase II residues. On the contrary, conjugates **1**–**5** were predicted to form a number of strong interactions with topoisomerase II protein residues, resulting in relatively high binding constants for all conjugates, approximately 1–2 orders of magnitude higher than the predicted Ki of DiMIQ (**6**). The highest Gibbs free energy of binding to both topoisomerase II isoforms was predicted for conjugate **2**. Overall, the in silico and in vitro data suggest that conjugate of ortho-coumaric acid **2** exhibited biological and chemical properties that warrant further biological research, both in vitro and in vivo, to prove its cytotoxic profile and the mechanism of action. In addition, this compound can be considered as the lead structure for further modifications to find selective conjugates of natural origin that can be used in the future to treat pancreatic cancers.

## 5. Patents

The structures of the compounds were claimed in a Polish Patent Application, p.445978

## Figures and Tables

**Table 1:** Calculated IC_50_ (half maximum inhibitory concentration) and SI (selectivity index) for **1** and **2** against pancreatic, cervical, and breast cancer lines, measured by the cell viability MTT assay.

Cell Line	SI	IC_50_ Compound 1	IC_50_ Compound 2	IC_50_ DiMIQ (6)
AsPC-1	-	2641.50 ± 286.66	336.50 ± 85.01	1622.50 ± 131.98
BxPC-3	-	805.00 ± 63.63	347.53 ± 52.39	888.77 ± 49.52
HeLa	-	808.75 ± 91.29	203.15 ± 35.28	784.20 ± 20.03
MCF-7	-	1904.00 ± 50.91	748.45 ± 12.23	868.30 ± 18.81
NHDF	-	2301.33 ± 157.85	1714.33 ± 53.01	2332.33 ± 354.03
-	AsPC-1	0.87	5.09	1.44
-	BxPC-3	2.86	4.93	2.62
-	HeLa	2.85	8.44	2.97
-	MCF-7	1.21	2.29	2.69

**Table 2:** Computationally estimated Gibbs free energy of binding and Ki values for investigated ligands and DNA model 1Z3F.

Ligand	Gibbs Free Energy of Binding [kcal/mol]	Ki [nM]
DiMIQ (6)	−7.80	1900
1	−8.76	380
2	−8.77	380
3	−8.59	510
4	−9.25	170
5	−8.34	770
ellipticine	−8.97	270
cryptolepine	−7.85	1800
levogatifloxacin	−7.48	3300
levofloxacin	−6.37	21,400
etoposide	−4.06	34,340,000

**Table 3:** Computationally estimated Gibbs free energy of binding (Gbind) and Ki values for investigated ligands and topoisomerase II alpha/beta complex with DNA.

Ligand	Topoisomerase II Alpha		Topoisomerase II Beta	
	G_bind_ [kcal/mol]	K_i_ [nM]	G_bind_ [kcal/mol]	K_i_ [nM]
DiMIQ (**6**)	−7.73	2160	−9.13	202.8
**1**	−10.26	29.9	−11.04	8.1
**2**	−10.64	15.9	−11.67	2.8
**3**	−10.68	14.9	−11.16	6.6
**4**	−10.16	35.8	−10.87	10.7
**5**	−9.85	60.5	−10.54	18.7
ellipticine	−8.93	282.6	−10.05	43.1
cryptolepine	−7.74	2120	−9.12	206.6
levogatifloxacin	−11.62	3.1	−10.73	13.6
levofloxacin	−10.03	44.6	−10.17	34.8
etoposide	−11.23	5.8	−10.51	309.3

## Data Availability

The datasets presented in the current study are available from the corresponding author on reasonable request.

## Supplementary Materials

The following supporting information can be downloaded at: https://www.mdpi.com/article/10.3390/ijms25052573/s1. ^1^H and 13C NMR spectra (Figures S1–S10), charts of potential toxicity (Figures S11–S16), predicted sites of CYP450 metabolism (Figure S17), stability of compounds in DMSO solutions (Figures S18 and S19), predicted ADMET properties (Tables S1 and S2).

## Abbreviations

HCA—hydroxycinnamic acids; DiMIQ—5,11-dimethyl-5*H*-indolo[2,3-*b*]quinoline; BxPC-3—pancreatic cancer cell line; AsPC-1—metastatic pancreatic cancer cell line; MCF-7—breast cancer cell line; HeLa—cervical cancer cell line; NHDF—the normal human dermal fibroblast cell line; GLUT—glucose transporter proteins; BPV 1—bovine papillomavirus type 1; MS—mass spectrometry; NMR—nuclear magnetic resonance; TMS—trimethylsilyl; s, d, t, q, m, bs—singlet, doublet, triplet, quartet, multiplet and broad singlet in NMR spectra; DIPEA—N,N–diisopropylethylamine; DMAP—4–dimethylaminopyridine; EDCI—1-Ethyl-3-(3-dimethylaminopropyl)carbodiimide; DCC—N,N′–dicyclohexylcarbodiimide; TBTU—O-(1H-benzotriazol-1-yl)-1,1,3,3-tetramethyluronium tetrafluoroborate; AcOEt—ethyl acetate; DCM—dichloromethane; DMF—N,N-dimethylformamide; MTT—3-(4,5–dimethylthiazol-2-yl)-2,5-diphenyltetrazolium bromide; ESI–MS—electrospray ionization; HRMS—high-resolution mass spectrometry; DMSO—dimethyl sulfoxide; DMEM—Dulbecco’s Modified Eagle Medium; alpha MEM—alpha Minimum Essential Medium; FBS—fetal bovine serum.
