# Supplementary material for: The Conjugates of Indolo[2,3-b]quinoline as Anti-Pancreatic Cancer Agents: Design, Synthesis, Molecular Docking and Biological Evaluations
Source: Int J Mol Sci. 2024 Feb 22;25(5):2573. doi: 10.3390/ijms25052573 (PMC10932360; doi:10.3390/ijms25052573)
Supplement: Supplementary file 1 [file ijms-25-02573-s001.zip › ijms-2871439-supplementary.pdf]

Supporting Information

The conjugates of indolo[2,3-*b*]quinoline as anti-pancreatic cancer agents: design, synthesis, molecular docking and biological evaluations

Marcin Cybulski [1], Katarzyna Sidoryk [1], Magdalena Zaremba-Czogalla [2], Bartosz Trzaskowski [3], Marek Kubiszewski [4], Joanna Tobiasz [1], Anna Jaromin [2] and Olga Michalak [1,*]

[1] Pharmacy, Cosmetic Chemistry and Biotechnology Research Group, Łukasiewicz Research Network-Industrial Chemistry Institute, Warsaw, Poland; marcin.cybulski@ichp.lukasiewicz.gov.pl (M.C.); k.sidoryk@pikralida.eu (K.S.); olga.michalak@ichp.lukasiewicz.gov.pl (O.M.)
[2] Department of Lipids and Liposomes, Faculty of Biotechnology, University of Wroclaw, Wroclaw, Poland; magdalena.zaremba-czogalla@uwr.edu.pl (M.Z.-C.); anna.jaromin@uwr.edu.pl (A.J.)
[3] Chemical and Biological Systems Simulation Lab, Center of New Technologies, University of Warsaw, Warsaw, Poland; b.trzaskowski@cent.uw.edu.pl
[4] Pharmaceutical Analysis Laboratory, Łukasiewicz Research Network-Industrial Chemistry Institute, Warsaw, Poland; marek.kubiszewski@ichp.lukasiewicz.gov.pl (M.K.)

*Correspondence: olga.michalak@ichp.lukasiewicz.gov.pl

In memory of Professor Łukasz S. Kaczmarek

TABLE OF CONTENTS

^{1}H and ^{13}C NMR spectra

Figure S1. ^{1}H and ^{13}C NMR spectra of 9-[((4-hydroxy)cinnamoyl)amino]-5,11-dimethyl-5H-indolo[2,3-*b*]quinoline (**1**)
Figure S2. ^{1}H and ^{13}C NMR spectra of 9-[((2-hydroxy)cinnamoyl)amino]-5,11-dimethyl-5*H*-indolo[2,3-*b*]quinoline (**2**)
Figure S3. ^{1}H and ^{13}C NMR spectra of 9-[((3-hydroxy)cinnamoyl)amino]-5,11-dimethyl-5*H*-indolo[2,3-*b*]quinoline (**3**)
Figure S4. ^{1}H and ^{13}C NMR spectra of 9-[(2,4-dihydroxy)cinnamoyl)amino]-5,11-dimethyl-5*H*-indolo[2,3-*b*]quinoline (**4**)
Figure S5. ^{1}H and ^{13}C NMR spectra of 9-[(3,4-dihydroxy)cinnamoyl)amino]-5,11-dimethyl-5*H*-indolo[2,3-*b*]quinoline (**5**)
Figure S6. ^{1}H and ^{13}C NMR spectra of 9-[((4-hydroxy)cinnamoyl)amino]-5,11-dimethyl-5*H*-indolo[2,3-*b*]quinoline (**1**) dihydrochloride
Figure S7. ^{1}H and ^{13}C NMR spectra of 9-[((2-hydroxy)cinnamoyl)amino]-5,11-dimethyl-5*H*-indolo[2,3-*b*]quinoline (**2**) dihydrochloride
Figure S8. ^{1}H and ^{13}C NMR spectra of 9-[((3-hydroxy)cinnamoyl)amino]-5,11-dimethyl-5*H*-indolo[2,3-*b*]quinoline (**3**) dihydrochloride
Figure S9. ^{1}H and ^{13}C NMR spectra of 9-[(2,4-dihydroxy)cinnamoyl)amino]-5,11-dimethyl-5*H*-indolo[2,3-*b*]quinoline (**4**) dihydrochloride

Figure S10. ^{1}H and ^{13}C NMR spectra of 9-[(3,4-dihydroxy)cinnamoyl)amino]-5,11-dimethyl-5H-indolo[2,3-b]quinoline (**5**) dihydrochloride

Computational studies

Table S1. Selected, predicted ADMET properties of compounds investigated in this work.
Table S2. Selected, predicted ADMET properties of compounds investigated in this work.
Figure S11. Chart of probabilities for activities related to potential toxocity for compound **1**
Figure S12. Chart of probabilities for activities related to potential toxocity for compound **2**
Figure S13. Chart of probabilities for activities related to potential toxocity for compound **3**
Figure S14. Chart of probabilities for activities related to potential toxocity for compound **4**
Figure S15. Chart of probabilities for activities related to potential toxocity for compound **5**
Figure S16. Chart of probabilities for activities related to potential toxocity for DiMIQ (**6**).
Figure S17. Predicted sites of CYP450 metabolism for compounds **1**-**5** and DiMIQ.

HPLC stability test

Figure S18. Stability of 9-[((4-hydroxy)cinnamoyl)amino]-5,11-dimethyl-5*H*-indolo[2,3-*b*]quinoline (**1**) dihydrochloride in DMSO solution at ambient temperature : **a)** at starting point "0" **b)** after 120 h

Figure S19. Stability of 9-[((2-hydroxy)cinnamoyl)amino]-5,11-dimethyl-5*H*-indolo[2,3-*b*]quinoline (**2**) dihydrochloride in DMSO solution at ambient temperature : **a)** at starting point "0" **b)** after 120 h

HPLC Metod: the chromatographic analysis was performed using a Waters HPLC system (Waters Assoc., Milford, MA, USA) consisting of two Waters pump 515, Waters autosampler 717-plus, Waters column oven and Photodiode Array Detector 2996. The Stationary phase – Symetry C-18 (250 × 4.6 mm, 5 μm). The mobile phase – A (0.1% TFA in acetonitrile) and B (0.1% TFA in water), elution – linear gradient, flow rate – 1 mL/min, duration time – 25 min.The other parameters: column temp. – 25°C, concentration of conjugates **1** and **2** – 0.05 mg/mL, injection volume – 40 μl, UV detection wavelength ʎ = 330 nm.

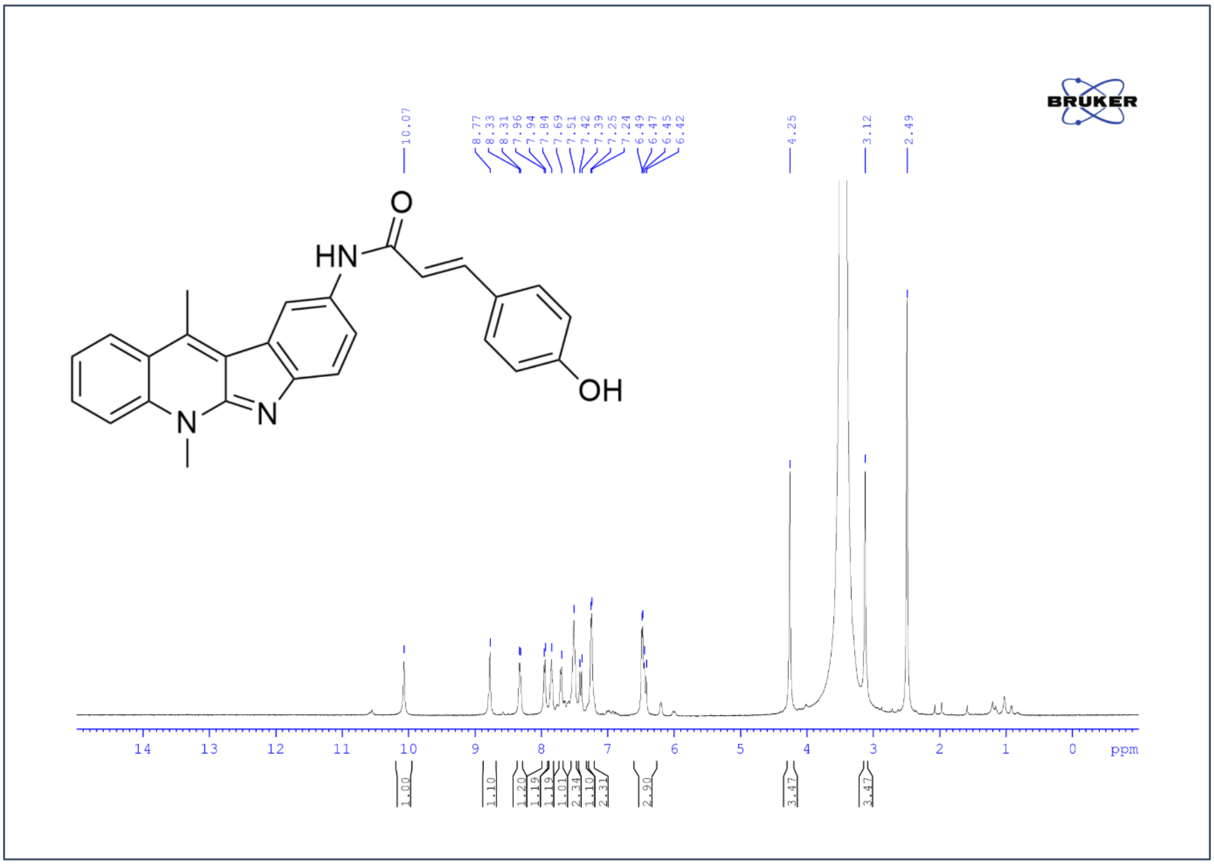

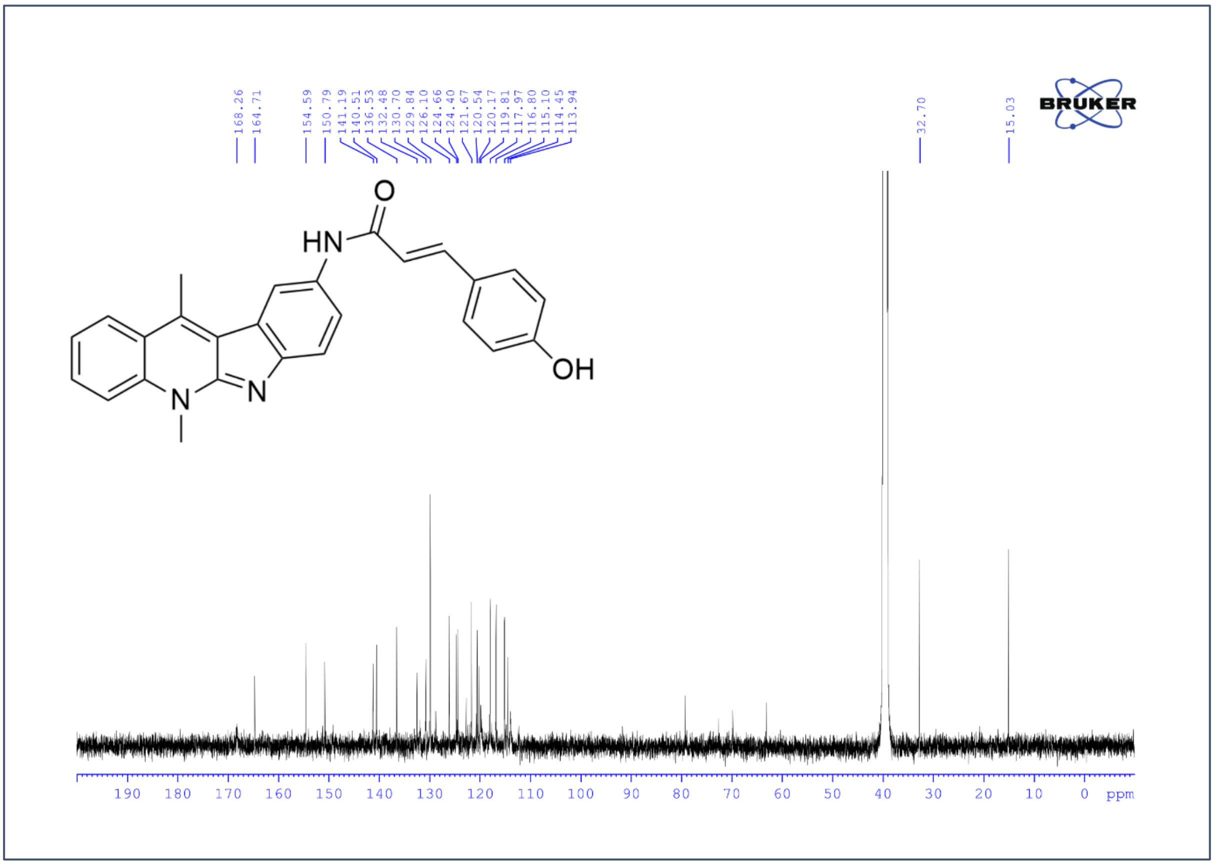

Figure S1. 9-[((4-hydroxy)cinnamoyl)amino]-5,11-dimethyl-5*H*-indolo[2,3-*b*]quinoline (**1**)

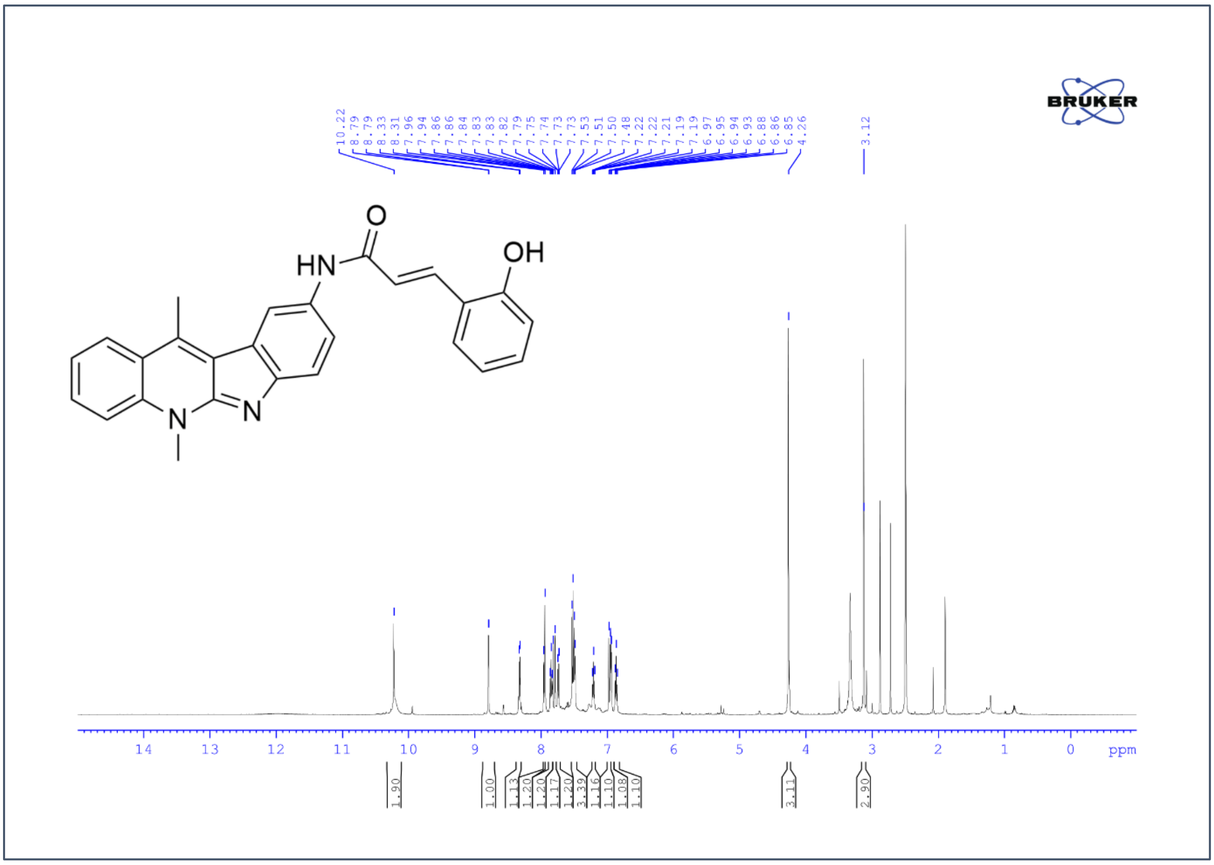

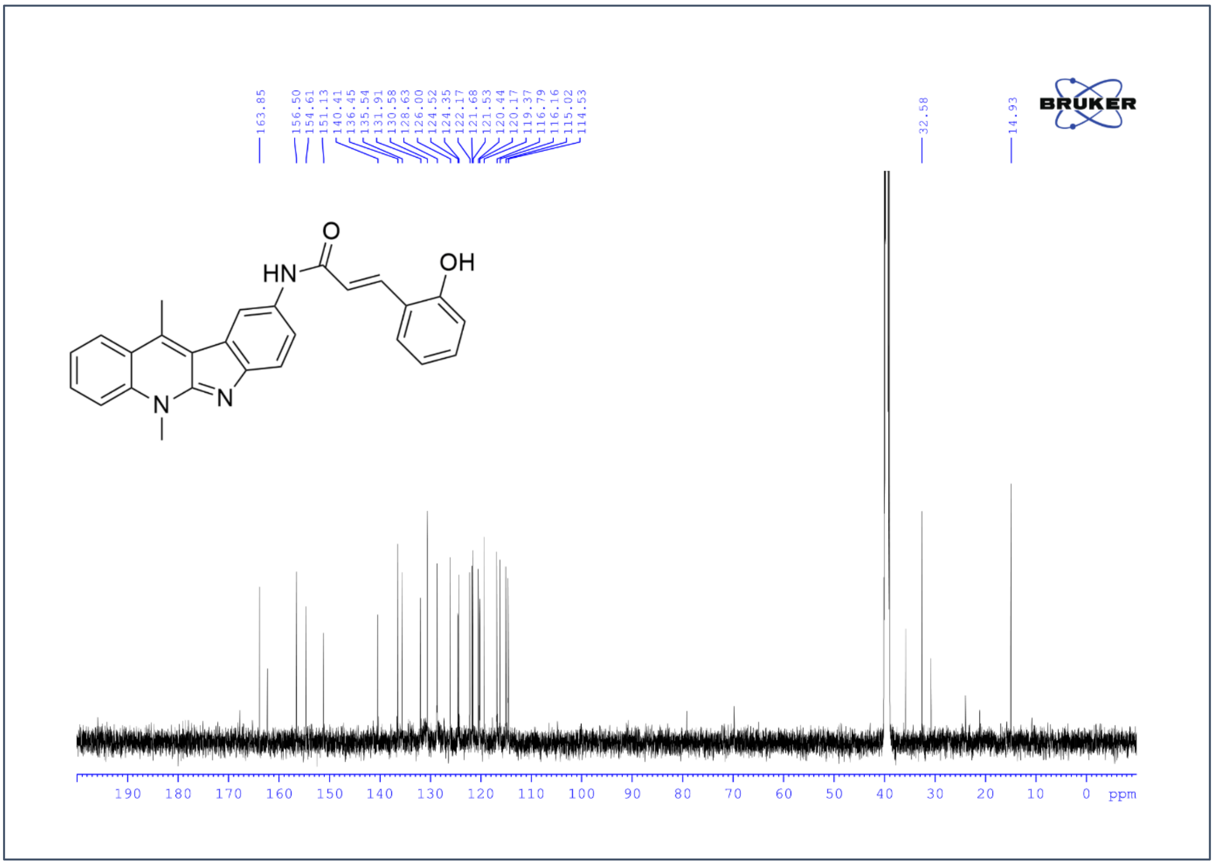

Figure S2. 9-[((2-hydroxy)cinnamoyl)amino]-5,11-dimethyl-5*H*-indolo[2,3-*b*]quinoline (**2**)

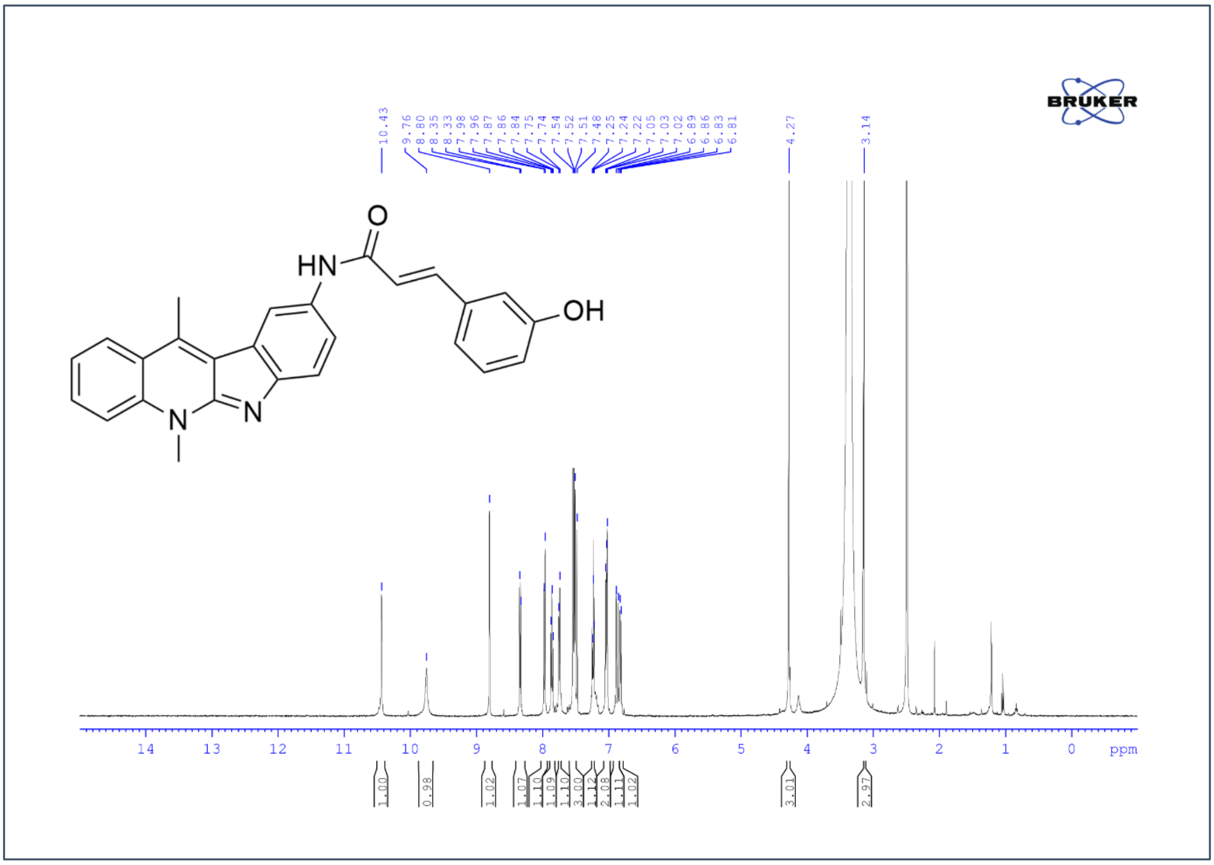

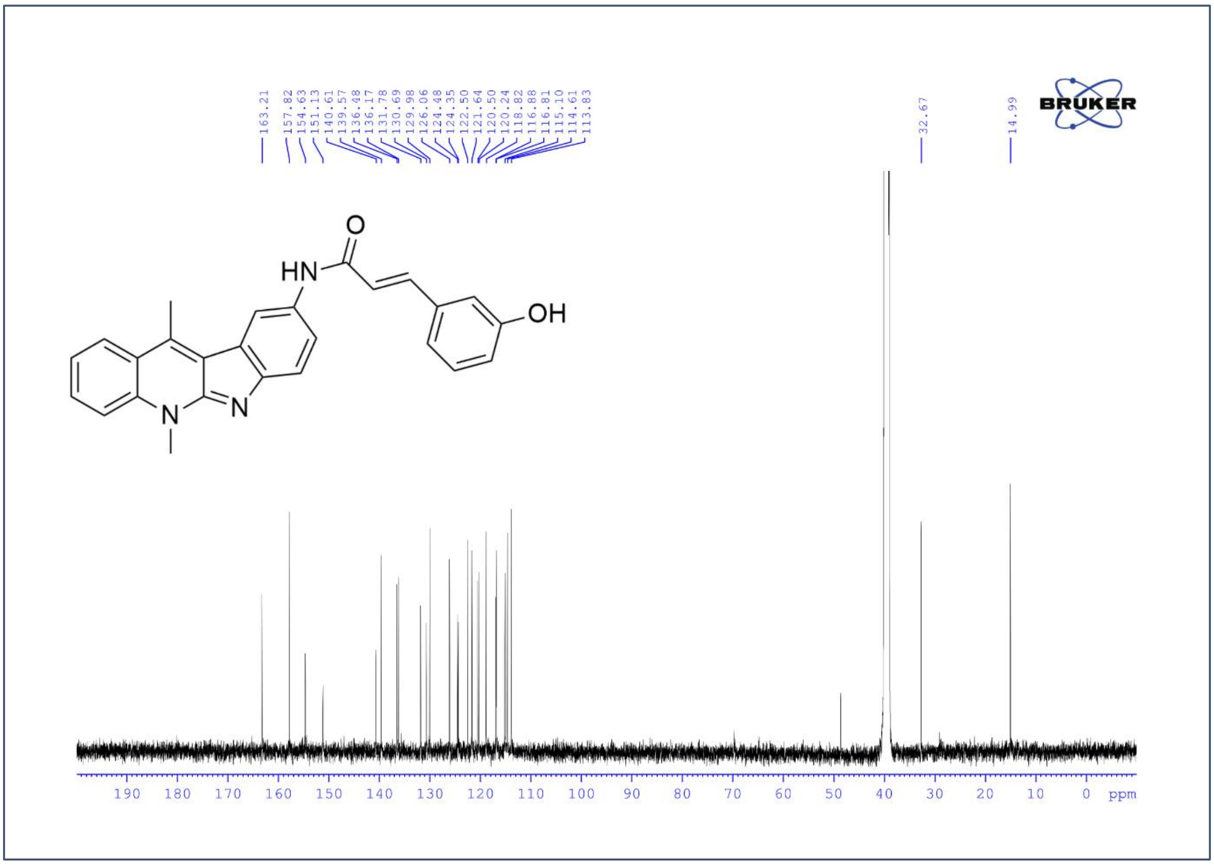

Figure S3. 9-[((3-hydroxy)cinnamoyl)amino]-5,11-dimethyl-5H-indolo[2,3-*b*]quinoline (**3**)

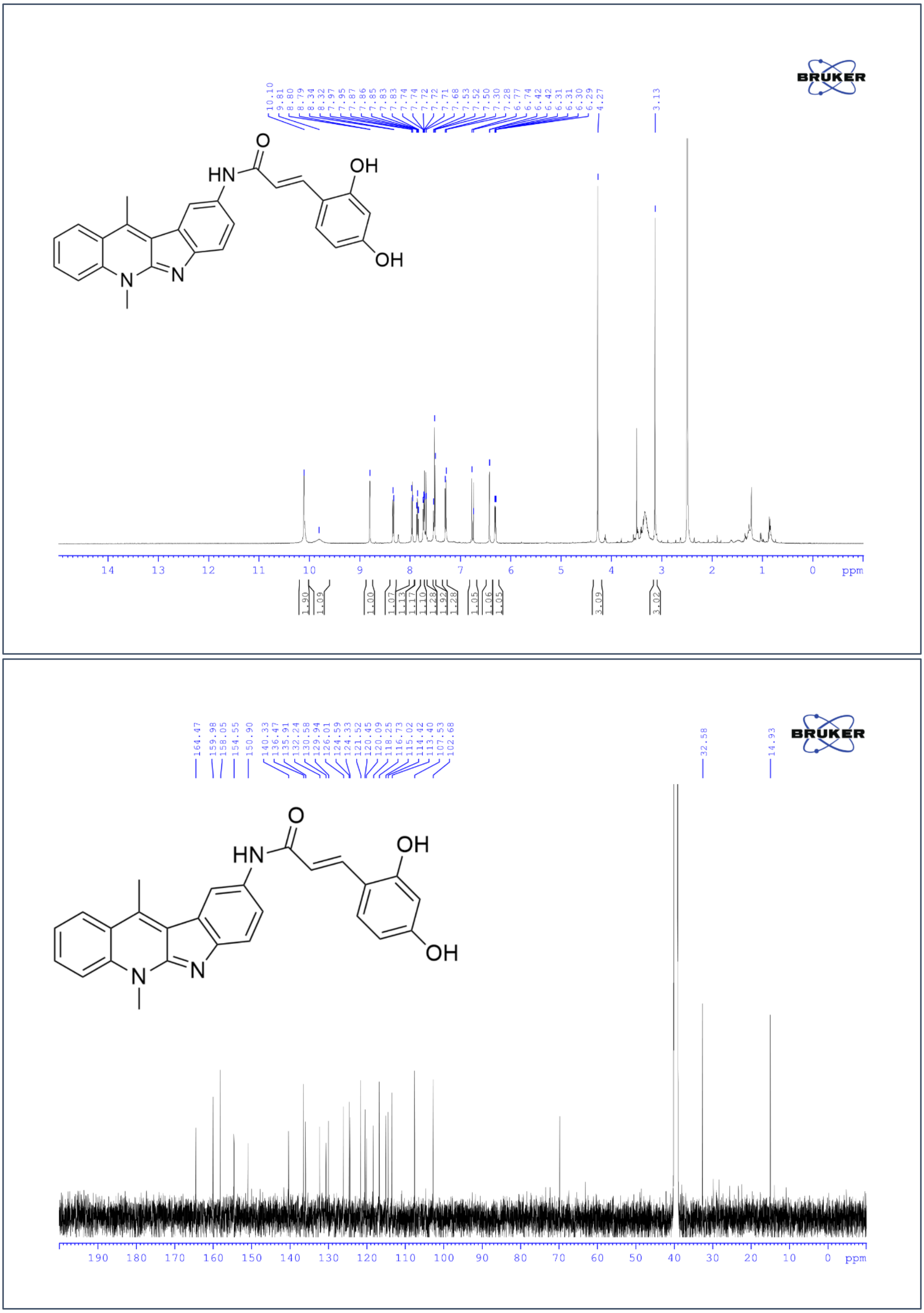

Figure S4. 9-[(2,4-dihydroxy)cinnamoyl)amino]-5,11-dimethyl-5*H*-indolo[2,3-*b*]quinoline (**4**)

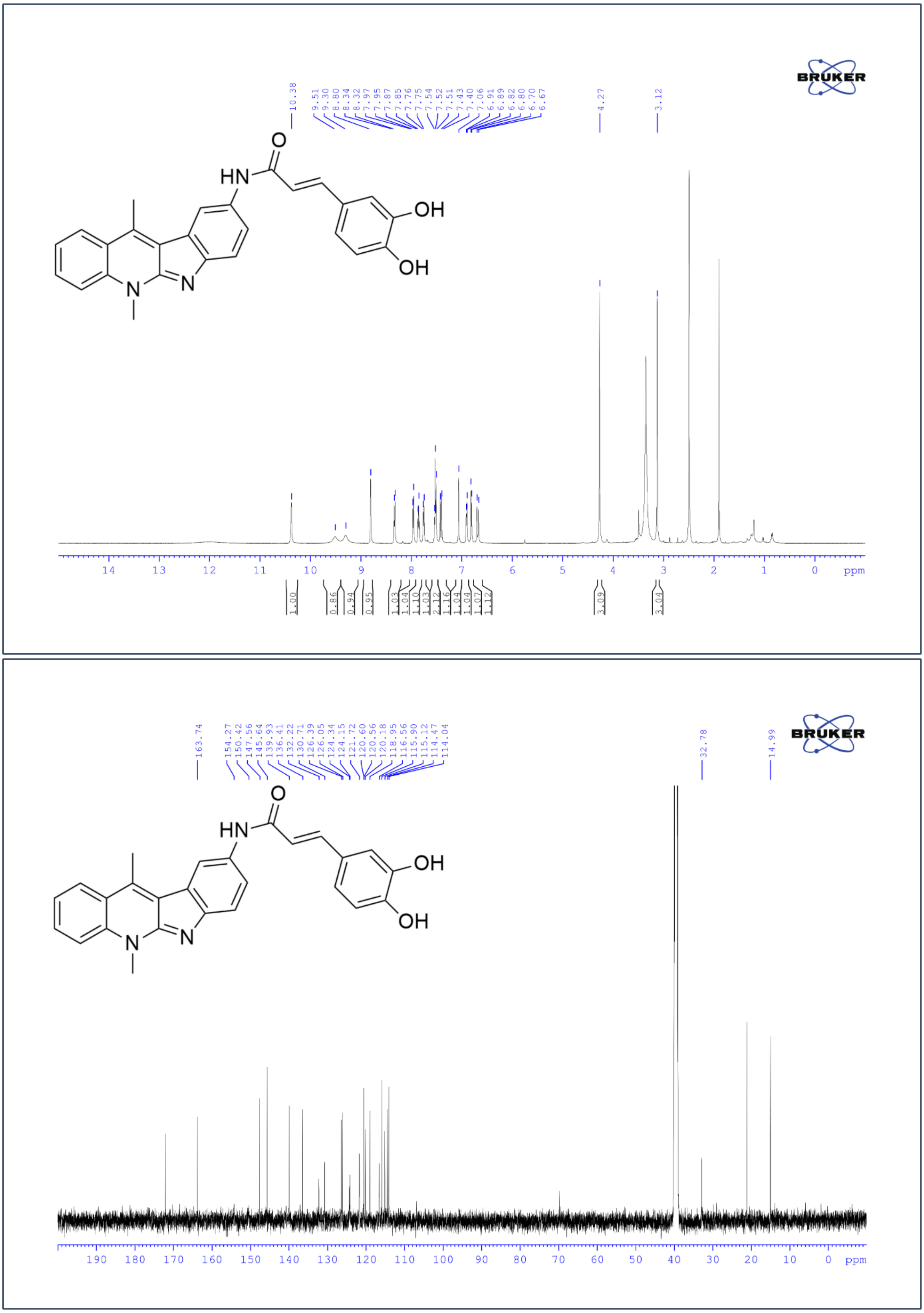

Figure S5. 9-[(3,4-dihydroxy)cinnamoyl)amino]-5,11-dimethyl-5*H*-indolo[2,3-*b*]quinoline (**5**)

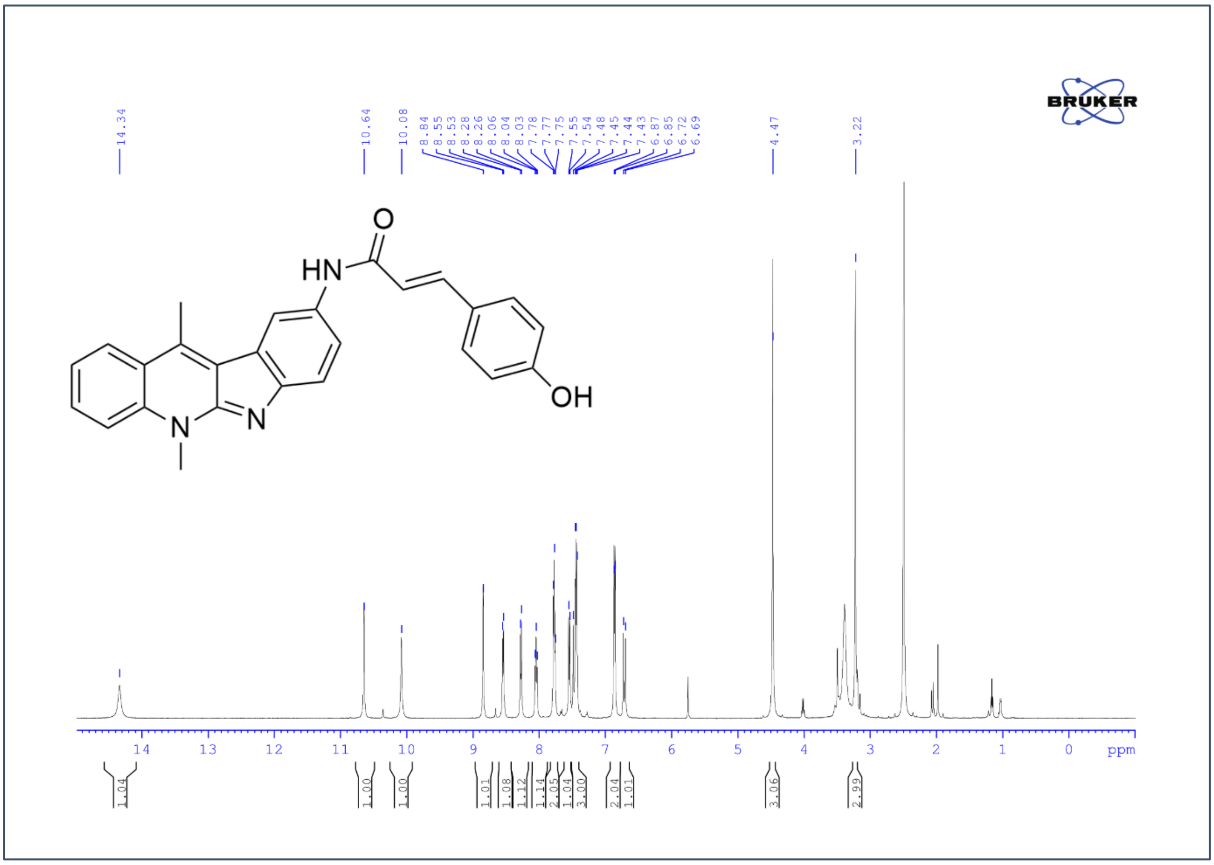

Figure S6. 9-[((4-hydroxy)cinnamoyl)amino]-5,11-dimethyl-5*H*-indolo[2,3-*b*]quinoline (**1**) dihydrochloride

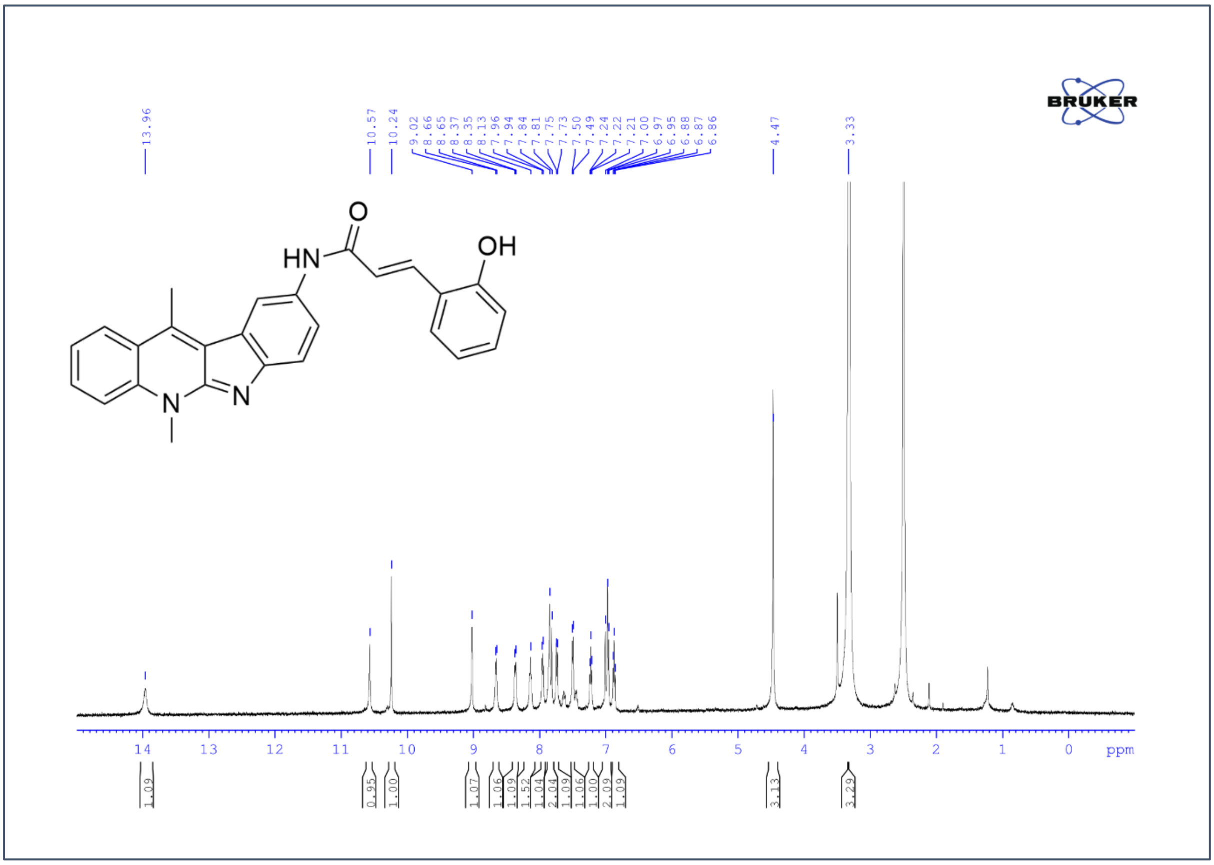

Figure S7. 9-[((2-hydroxy)cinnamoyl)amino]-5,11-dimethyl-5*H*-indolo[2,3-*b*]quinoline (**2**) dihydrochloride

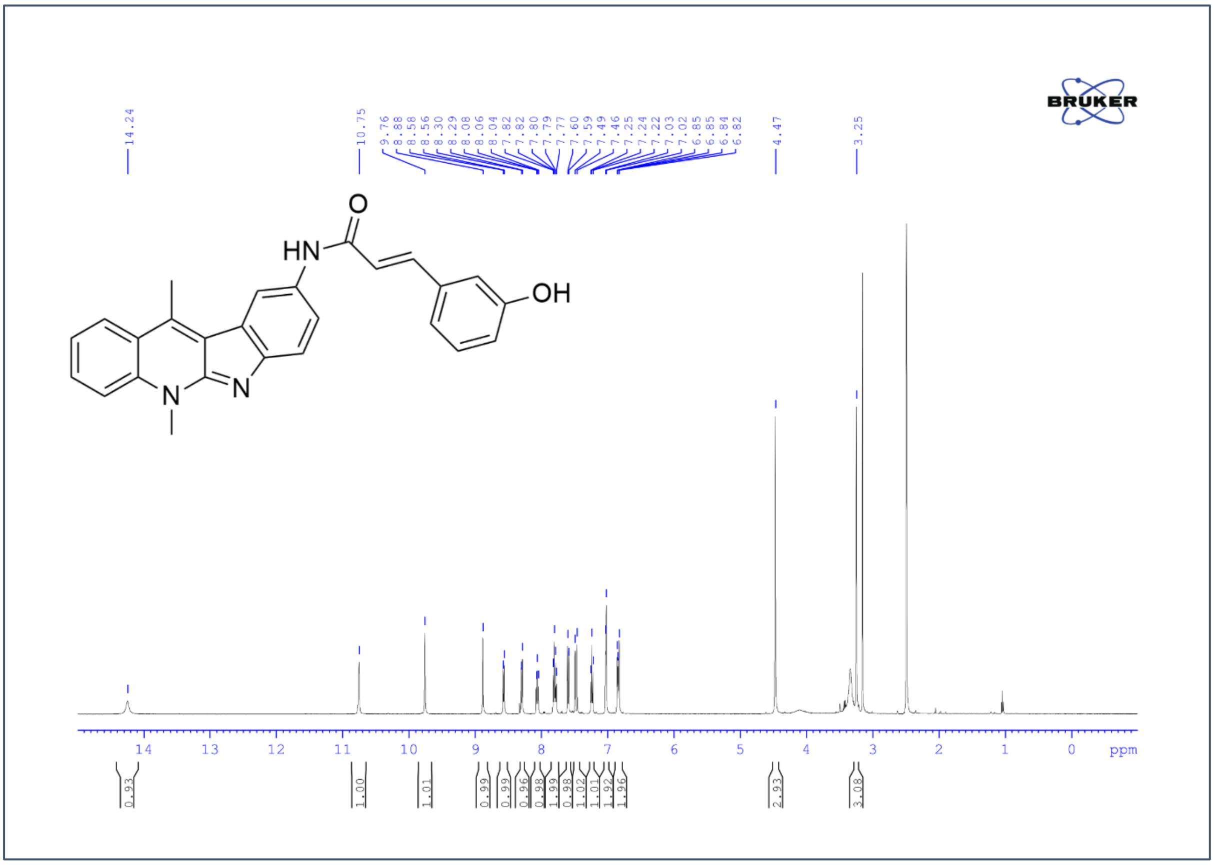

Figure S8. 9-[((3-hydroxy)cinnamoyl)amino]-5,11-dimethyl-5*H*-indolo[2,3-*b*]quinoline (**3**) dihydrochloride

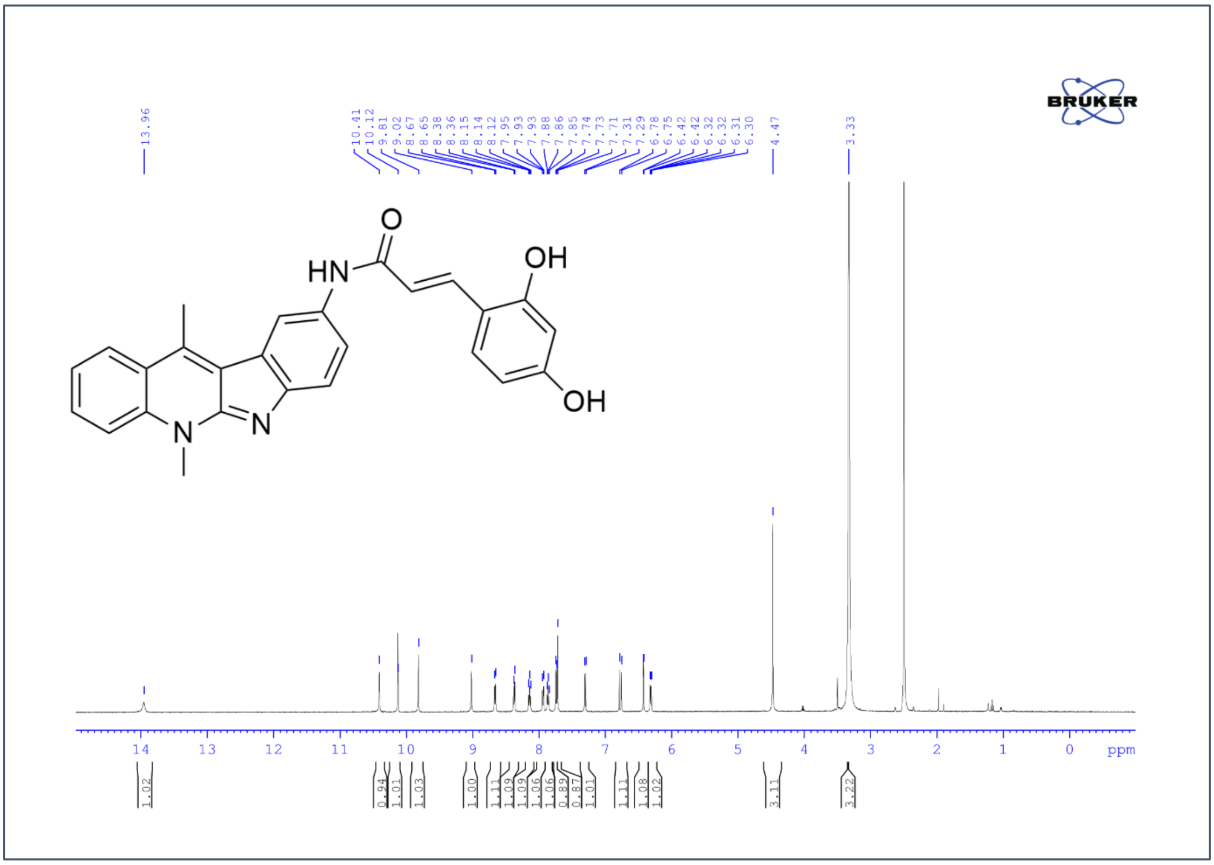

Figure S9. 9-[(2,4-dihydroxy)cinnamoyl)amino]-5,11-dimethyl-5*H*-indolo[2,3-*b*]quinoline (**4**) dihydrochloride

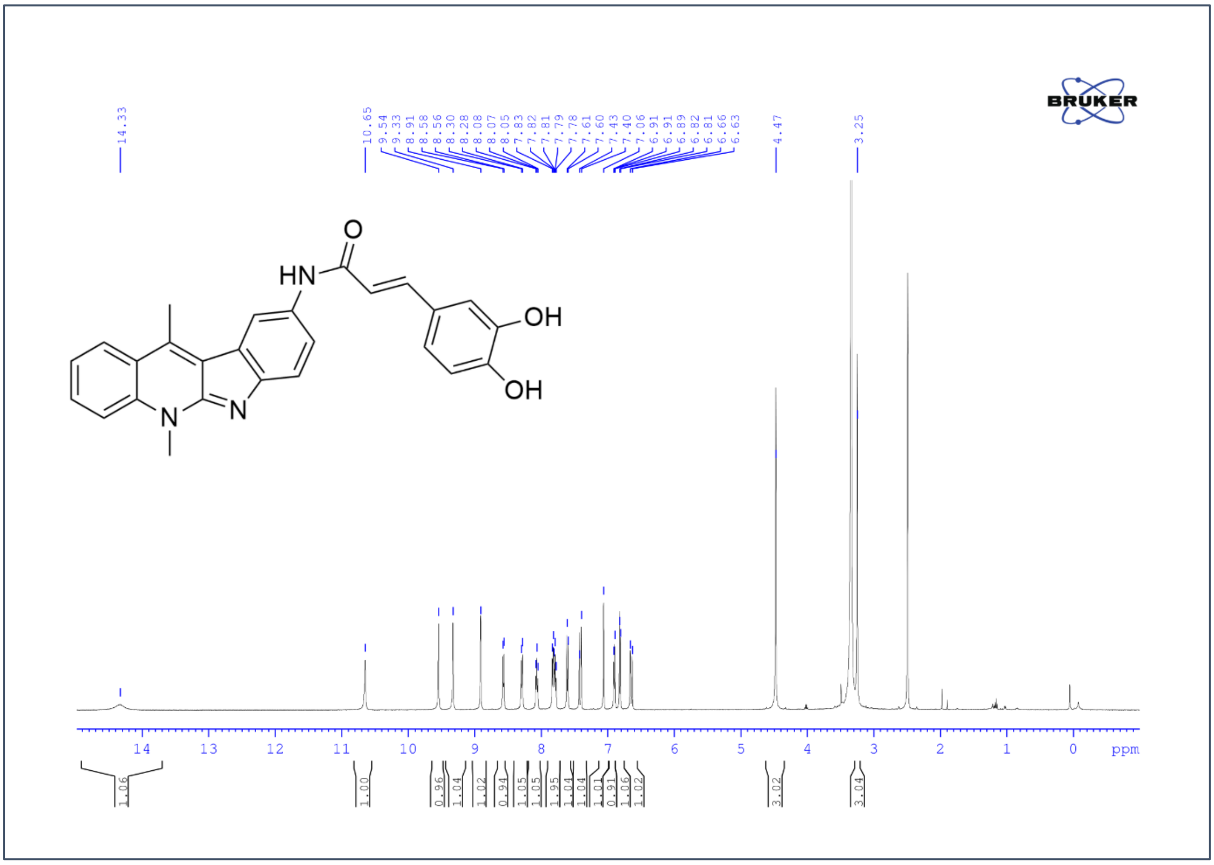

Figure S10. 9-[(3,4-dihydroxy)cinnamoyl)amino]-5,11-dimethyl-5*H*-indolo[2,3-*b*]quinoline (**5**) dihydrochloride

Table S1. Selected, predicted ADMET properties of compounds investigated in this work.

compound	MW[a]	dipole[b]	vol[c]	SASA[d]	dHB[e]	aHB[f]	logP[g]	logS[h]	metab[i]	P[j]	Ro3[k]	Ro5[l]
**1**	407.5	12.7	1271.7	711.1	2	5.25	4.40	-6.37	3	435	1	0
**2**	407.5	13.2	1267.6	707.8	2	5.25	4.46	-6.31	3	534	1	0
**3**	407.5	12.0	1271.7	711.1	2	5.25	4.40	-6.37	3	435	1	0
**4**	423.5	8.7	1290.6	720.2	3	6	3.69	-5.99	4	163	1	0
**5**	423.5	10.5	1292.7	721.9	3	6	3.69	-6.01	4	158	1	0
**DiMIQ (6)**	246.3	8.4	815.1	474.1	0	2	3.89	-4.15	2	6597	0	0

[a]MW – molecular weight (Da); [b]dipole – dipole moment (D); [c]vol – total molecular volume (Å^3); [d]SASA – solvent accessible surface (Å^2); [e]dHB – estimated number of hydrogen bonds that would be donated by the solute to water molecules in an aqueous solution; [f]aHB – estimated number of hydrogen bonds that would be accepted by the solute from water molecules in an aqueous solution; [g]logP – octanol/water partition coefficient; [g]logS – predicted aqueous solubility (mol/dm^3); [i]metab – number of likely metabolic reactions; [j]P – apparent Caco-2 permeability (nm/sec); [k]Ro3 – number of violations of Jorgensen's rule of three; [l]Ro5 – number of violations of Lipinski's rule of five.

Table S2. Selected, predicted ADMET properties of compounds investigated in this work.

compound	FOSA[a]	FISA[b]	PISA[c]	glob[d]	HERG[e]	BB[f]	MDCK[g]	Kp[h]	HSA[i]	Jm[j]	LD_{50}[k]
**1**	146.1	143.1	421.9	0.80	-6.81	-1.24	201.4	-2.19	0.78	0.001	680
**2**	141.8	133.7	432.3	0.80	-6.82	-1.13	251.4	-1.98	0.77	0.002	680
**3**	146.1	143.2	421.9	0.80	-6.81	-1.24	201.0	-2.19	0.78	0.001	680
**4**	141.8	188.1	390.4	0.80	-6.70	-1.78	69.7	-3.03	0.58	0.000	680
**5**	146.1	189.6	386.2	0.79	-6.69	-1.80	67.2	-3.08	0.58	0.000	10000
**DiMIQ (6)**	132.7	18.6	322.8	0.89	-4.85	0.41	3801.3	-0.73	0.45	3.252	330

[a]FOSA – hydrophobic component of the SASA; [b]FISA – hydrophilic component of the SASA; [c]PISA – π (carbon and attached hydrogen) component of the SASA; [d]glob – globularity descriptor; [e]HERG – predicted IC_{50} value for blockage of HERG K^+ channels; [f]BB – predicted brain/blood partition coefficient; [g]MDCK – predicted apparent MDCK cell permeability (nm/sec); [g]K_p – predicted skin permeability; [i]HSA – prediction of binding to human serum albumin; [j]J_m – Predicted maximum transdermal transport rate (μg cm^{-2} hr^{-1}); [k]LD_{50} – predicted value of median lethal dose (mg/kg).

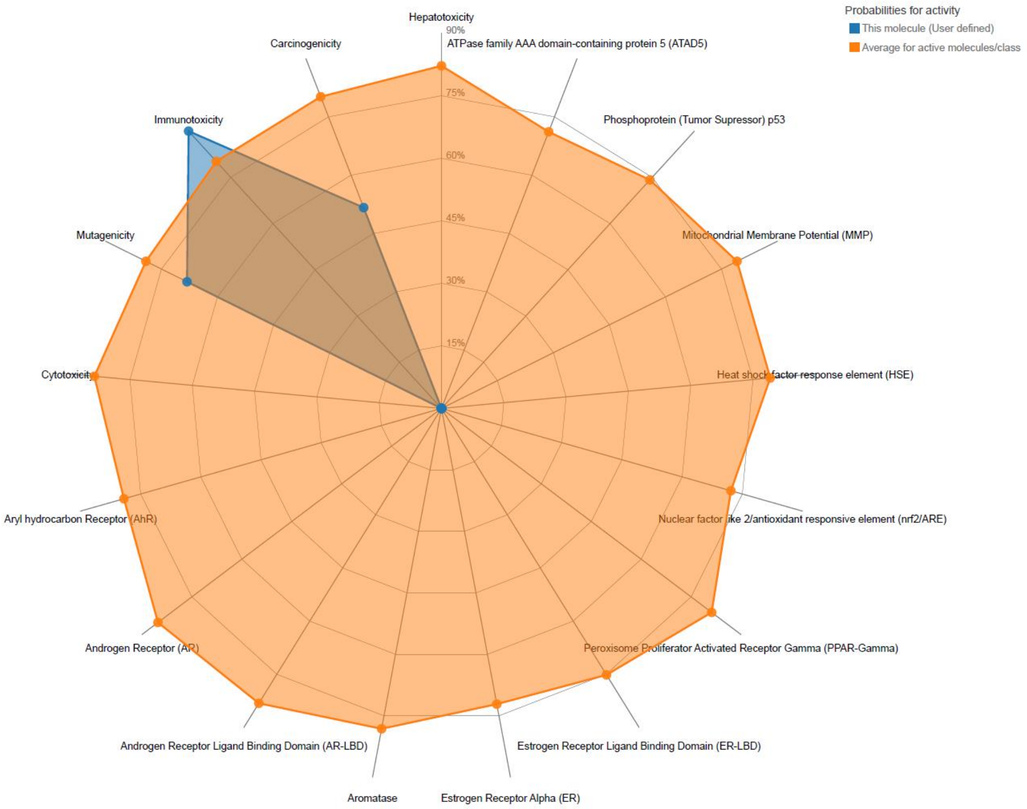

Figure S11. Chart of probabilities for activities related to potential toxocity for compound **1**.

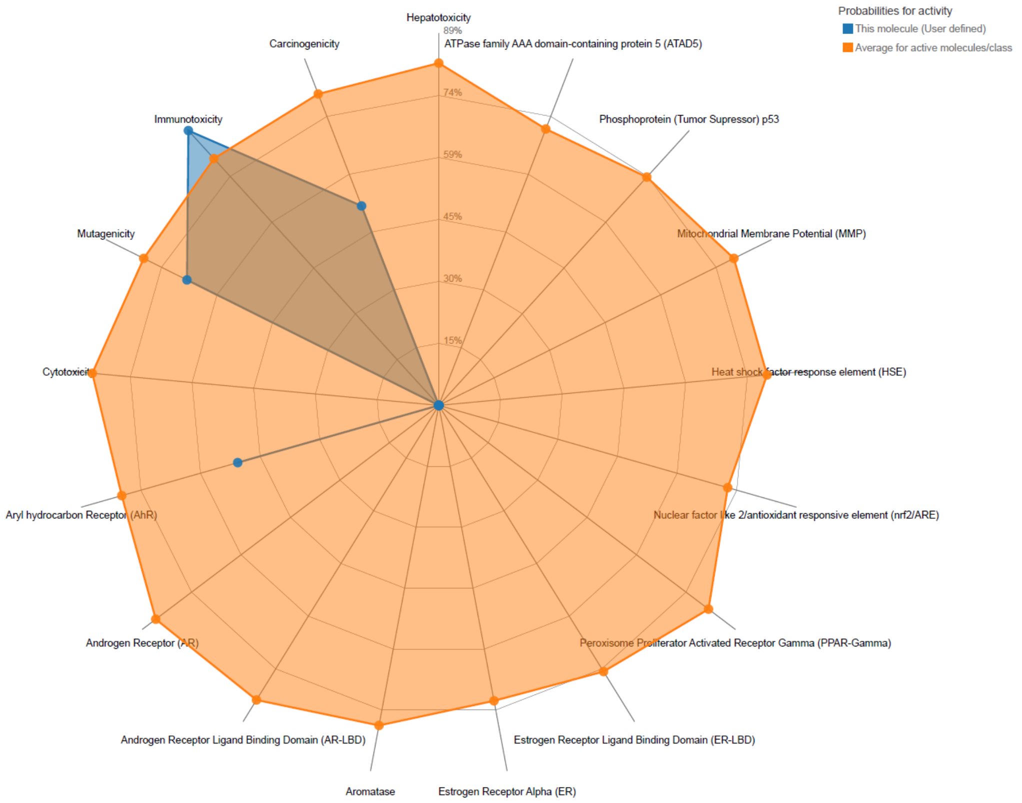

Figure S12. Chart of probabilities for activities related to potential toxocity for compound **2**.

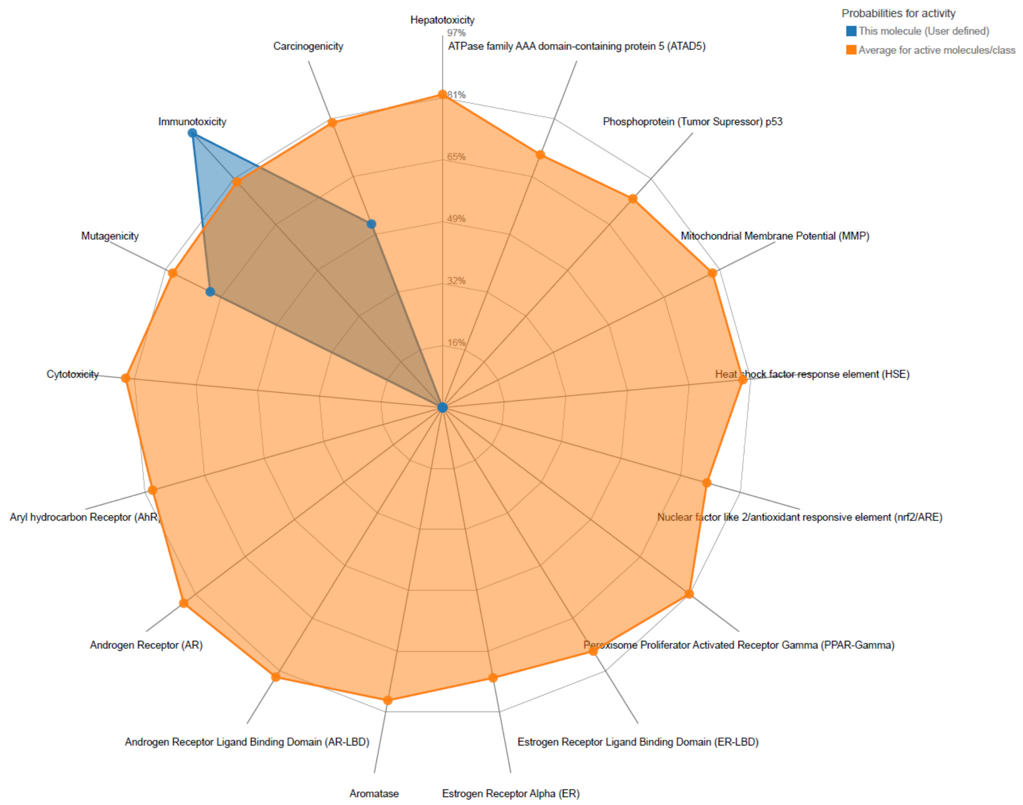

Figure S13. Chart of probabilities for activities related to potential toxocity for compound **3**.

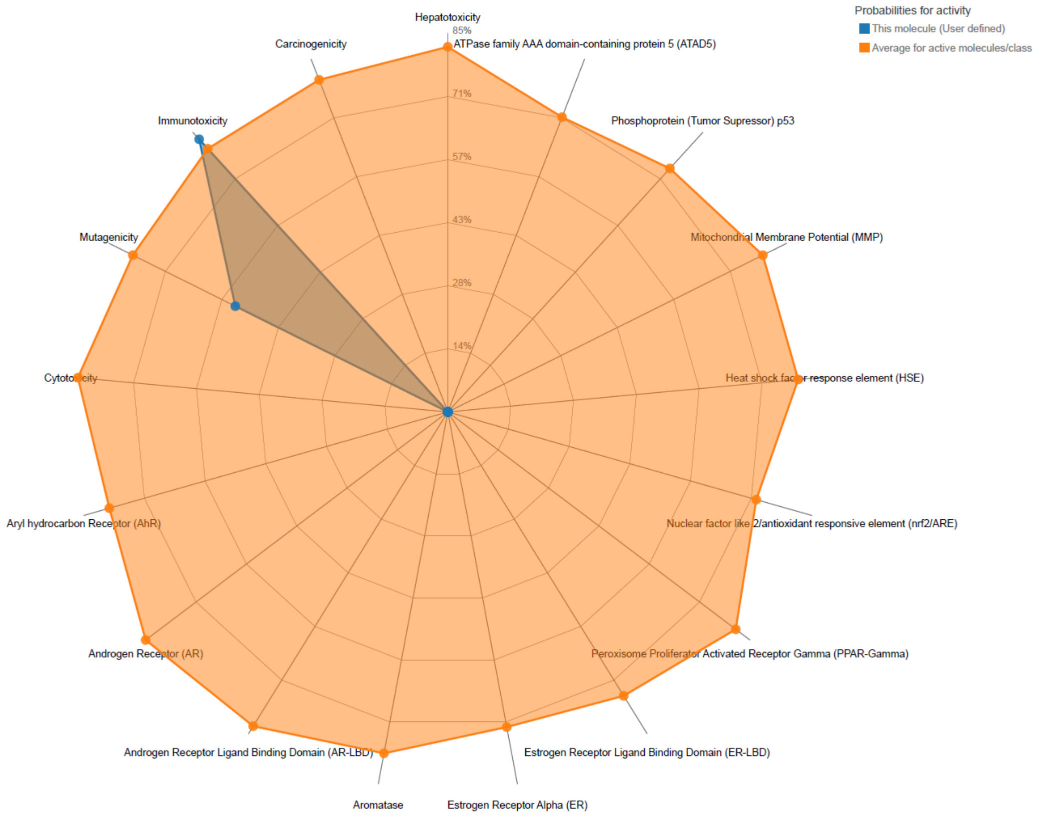

Figure S14. Chart of probabilities for activities related to potential toxocity for compound **4**.

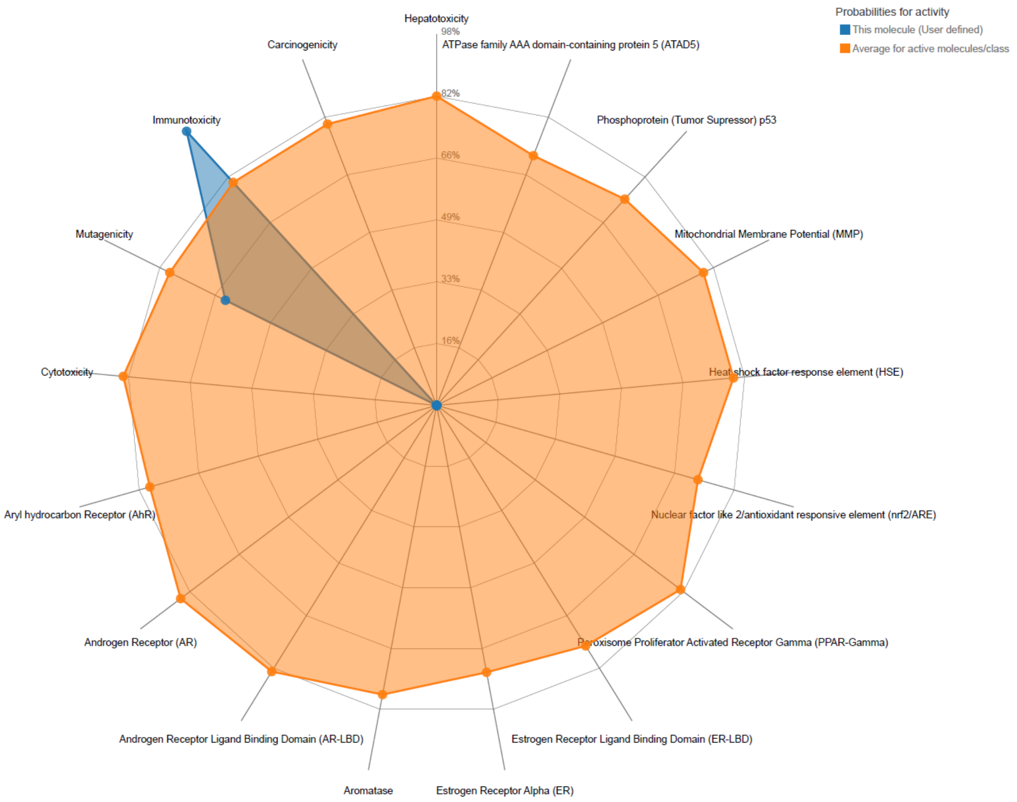

Figure S15. Chart of probabilities for activities related to potential toxocity for compound **5**.

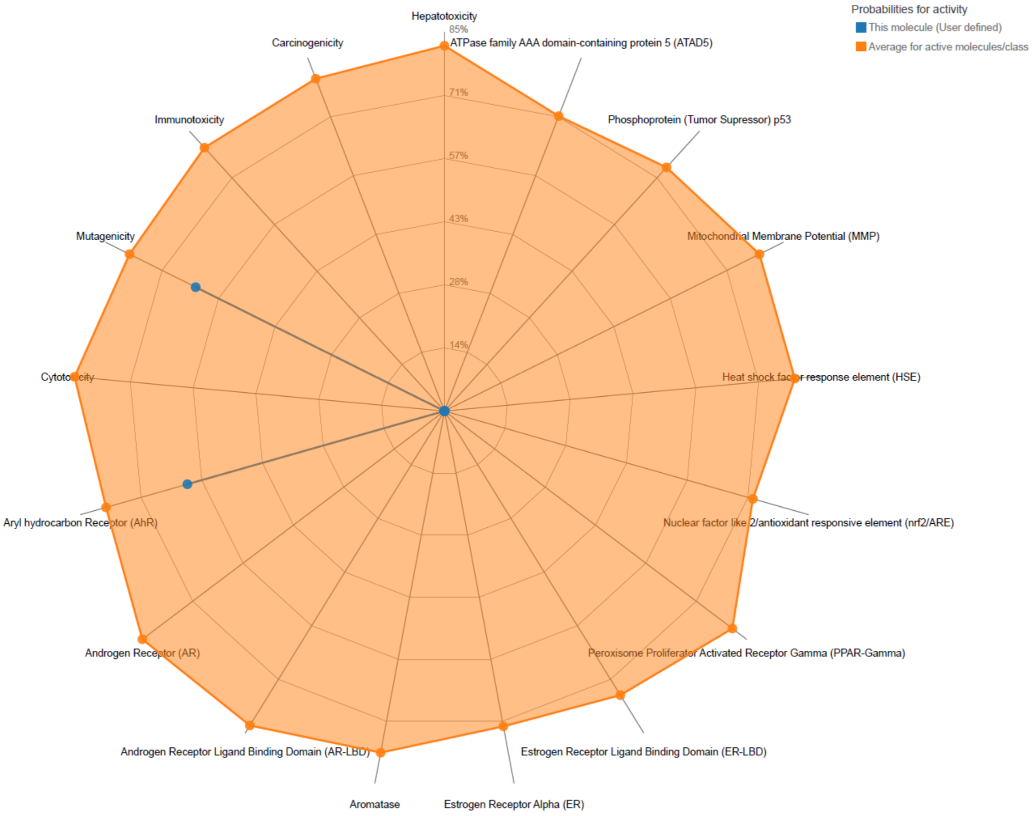

Figure S16. Chart of probabilities for activities related to potential toxocity for DiMIQ.

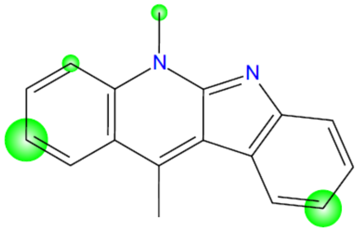

DiMIQ

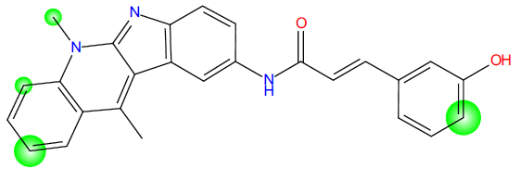

3

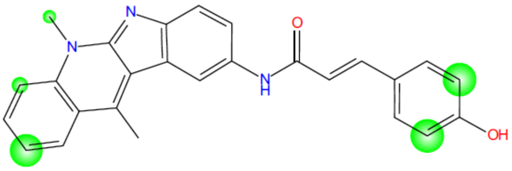

1

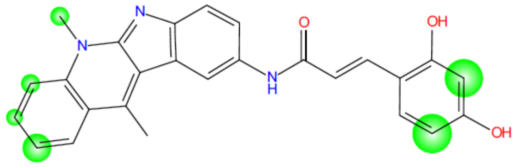

4

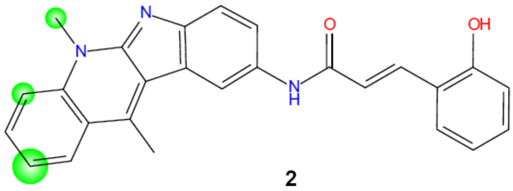

2

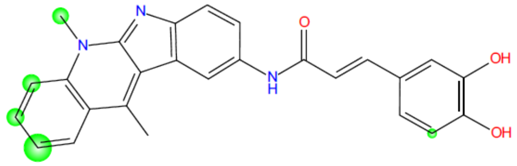

5

Figure S17. Predicted sites of CYP450 metabolism for compounds **1-5** and DiMIQ.

SAMPLE INFORMATION

Sample Name:	Conjugate 1 HCl DMSO "0" (3)	Acquired By:	tobiaszj
Sample Type:	Unknown		
Vial:	8	Acq. Method Set:	Indolochinoliny
Injection #:	1		
Injection Volume:	40,00 ul	Processing Method:	Indolochinoliny Con 1
Run Time:	25,0 Minutes	Channel Name:	330 nm
		Proc. Chnl. Descr.:	[PDA 330,0 nm] - Labeled: Blank[330] (PDA 190.0 to 800.0 nm at 1.2 nm)

Auto-Scaled Chromatogram

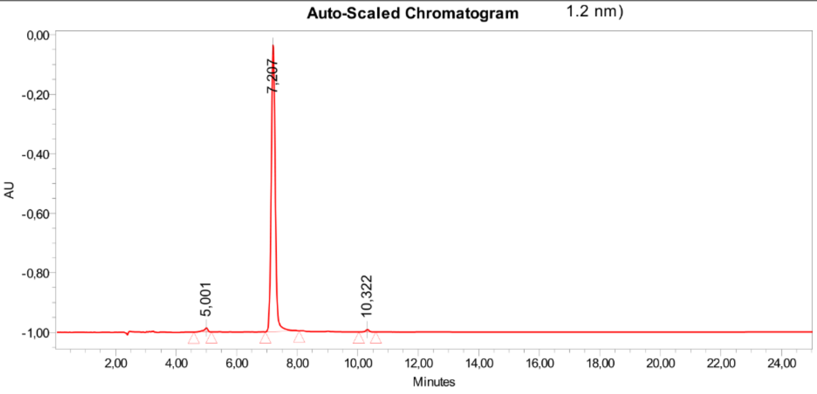

Peak Results

	Name	RT	Area	% Area	Symmetry Factor	Baseline Start (min)	Baseline End (min)	Selectivity	Resolution
1		5,001	139651	1,52	0,682992	4,583	5,167		
2		7,207	8966050	97,78	1,069331	6,950	8,067	1,551	9,734574
3		10,322	64042	0,70	0,918081	10,033	10,600	1,502	13,876856

a)

SAMPLE INFORMATION

Sample Name:	Conjugate 1 HCl DMSO 120h RT (3)	Acquired By:	tobiaszj
Sample Type:	Unknown		
Vial:	4	Acq. Method Set:	Indolochinoliny
Injection #:	1		
Injection Volume:	40,00 ul	Processing Method:	Indolochinoliny Con 1
Run Time:	25,0 Minutes	Channel Name:	330 nm
		Proc. Chnl. Descr.:	PDA 330,0 nm (PDA 190.0 to 800.0 nm at 1.2 nm)

Auto-Scaled Chromatogram

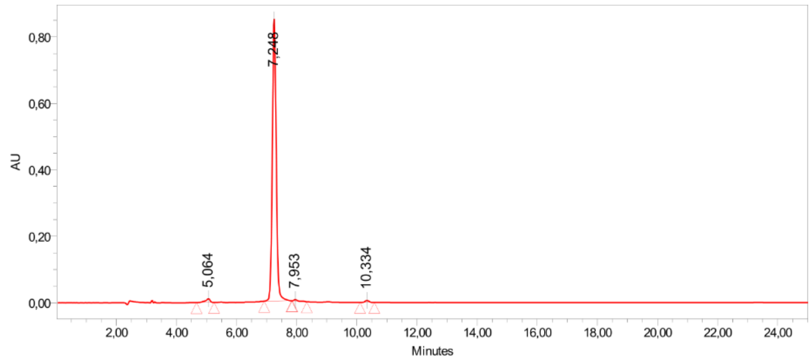

Peak Results

	Name	RT	Area	% Area	Symmetry Factor	Baseline Start (min)	Baseline End (min)	Selectivity	Resolution
1		5,064	122377	1,55	0,701518	4,667	5,250		
2		7,248	7670360	97,36	1,077603	6,917	7,833	1,537	9,148555
3		7,953	31429	0,40	2,347202	7,850	8,333	1,113	3,040656
4		10,334	54244	0,69	0,995788	10,100	10,583	1,343	10,271641

b)

Figure S18. Stability of 9-[((4-hydroxy)cinnamoyl)amino]-5,11-dimethyl-5*H*-indolo[2,3-*b*]quinoline (**1**) dihydrochloride in DMSO solution at ambient temperature : **a)** at starting point "0" **b)** after 120 h

SAMPLE INFORMATION

Sample Name:	Conjugate 2 HCl DMSO "0" (2)	Acquired By:	tobiaszj
Sample Type:	Unknown		
Vial:	13	Acq. Method Set:	Indolochinoliny
Injection #:	1		
Injection Volume:	40,00 ul	Processing Method:	Indolochinoliny Con 2
Run Time:	25,0 Minutes	Channel Name:	330 nm
		Proc. Chnl. Descr.:	PDA 330,0 nm (PDA 190.0 to 800.0 nm at 1.2 nm)

Auto-Scaled Chromatogram

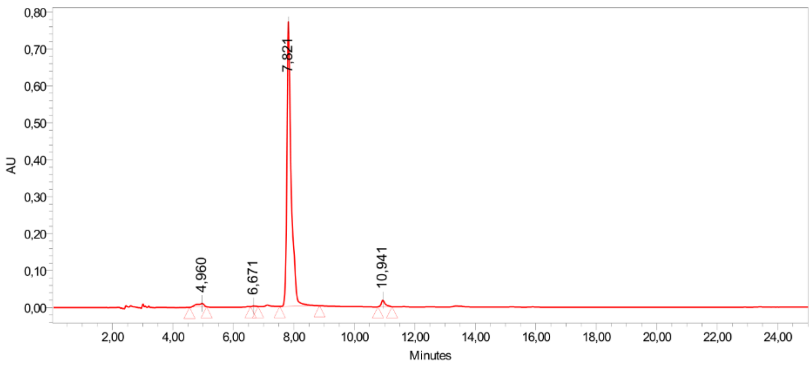

Peak Results

	Name	RT	Area	% Area	Symmetry Factor	Baseline Start (min)	Baseline End (min)	Selectivity	Resolution
1		4,960	174032	2,20	0,685754	4,550	5,117		
2		6,671	9794	0,12	1,278447	6,567	6,817	1,432	4,553160
3		7,821	7568744	95,59	1,592467	7,533	8,850	1,203	5,772222
4		10,941	165354	2,09	1,536011	10,783	11,250	1,457	14,560164

a)

SAMPLE INFORMATION

Sample Name:	Conjugate 2 HCl DMSO 120h RT (2)	Acquired By:	tobiaszj
Sample Type:	Unknown		
Vial:	5	Acq. Method Set:	Indolochinoliny
Injection #:	1		
Injection Volume:	40,00 ul	Processing Method:	Indolochinoliny Con 2
Run Time:	25,0 Minutes	Channel Name:	330 nm
		Proc. Chnl. Descr.:	PDA 330,0 nm (PDA 190.0 to 800.0 nm at 1.2 nm)

Auto-Scaled Chromatogram

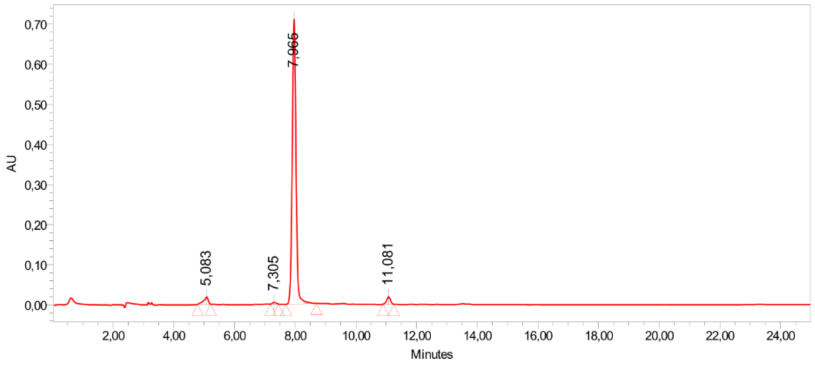

Peak Results

	Name	RT	Area	% Area	Symmetry Factor	Baseline Start (min)	Baseline End (min)	Selectivity	Resolution
1		5,083	183802	2,81	0,708198	4,783	5,217		
2		7,305	35293	0,54	1,118553	7,183	7,450	1,544	9,322693
3		7,965	6165959	94,22	1,016905	7,700	8,700	1,105	3,014682
4		11,081	159193	2,43	0,940313	10,900	11,250	1,447	13,877319

b)

Figure S19. Stability of 9-[((2-hydroxy)cinnamoyl)amino]-5,11-dimethyl-5*H*-indolo[2,3-*b*]quinoline (**2**) dihydrochloride in DMSO solution at ambient temperature : **a)** at starting point “0” **b)** after 120 h
